# Supplementary figures and images for: A phylogenetic study of the members of the MAPK and MEK families across Viridiplantae
Source: PLoS One. 2021 Apr 23;16(4):e0250584. doi: 10.1371/journal.pone.0250584 (PMC8064577; doi:10.1371/journal.pone.0250584)

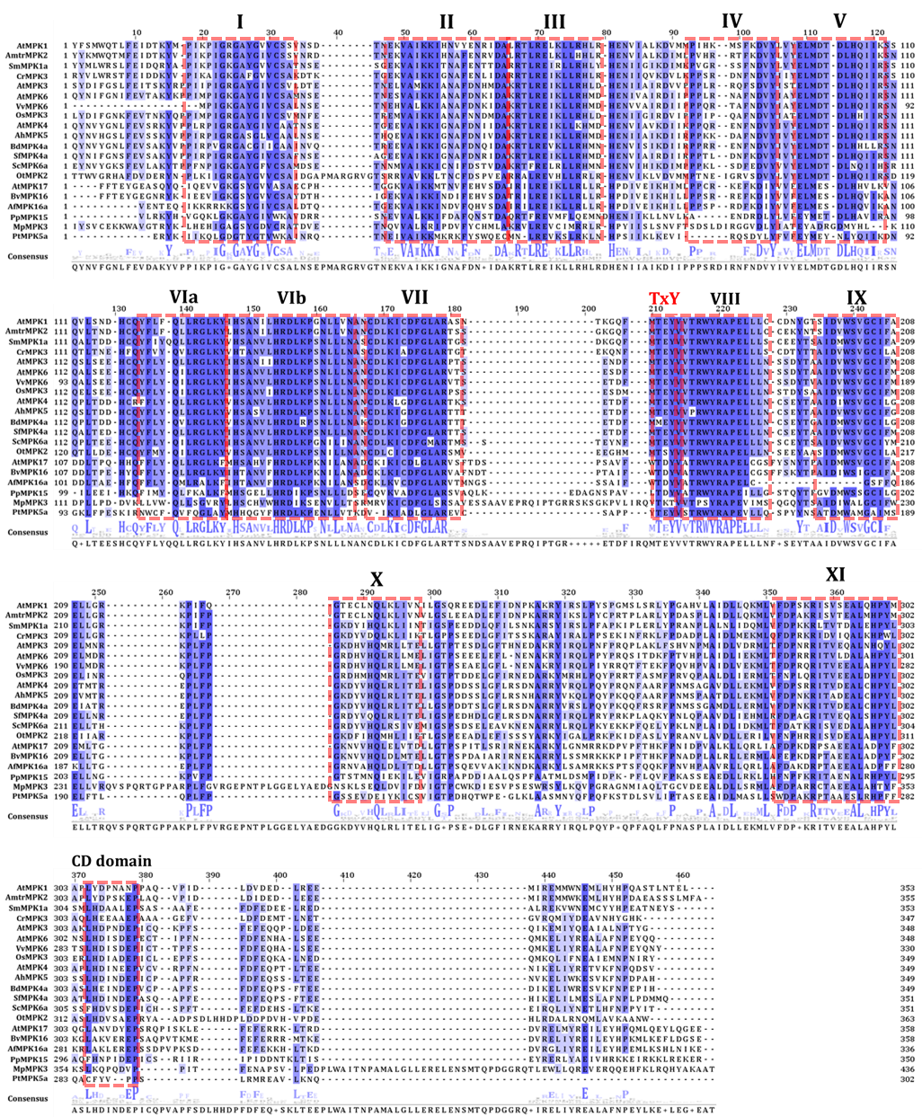

Supplement: S1 Fig — Twenty proteins representing the diversity of MAPKs in Viridiplantae were aligned using the MAFFT software [39] and visualized in Jalview [40]. The numerals I to XI indicate the conserved (canonical) kinase domains; TXY and CD labels indicate the activation site and docking domain, respectively. (TIF) [file pone.0250584.s001.tif]

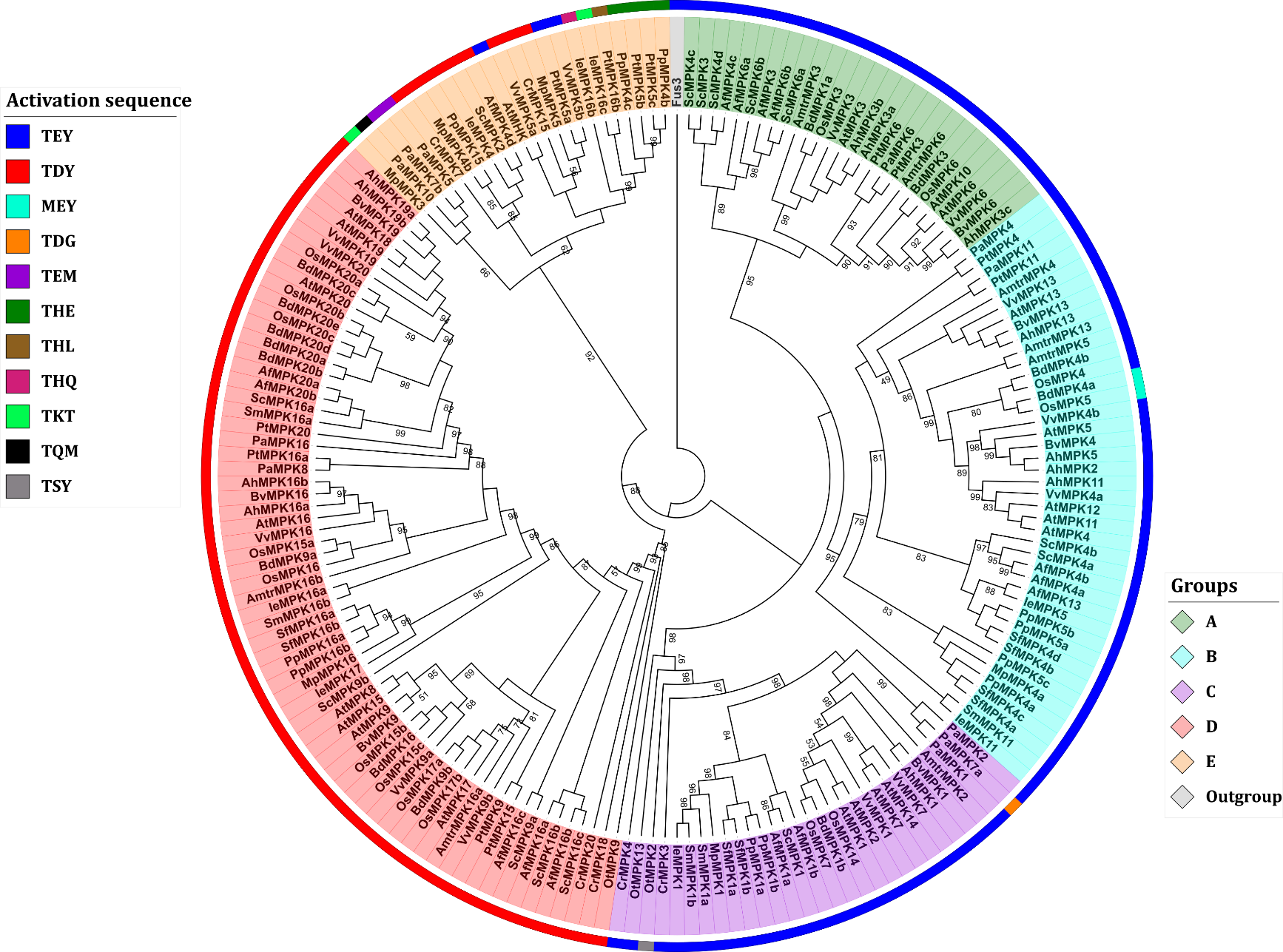

Supplement: S2 Fig — Most proteins from the A, B, and C group contain TEY activation motif, while proteins from group D contain the TDY activation motif. Group E exhibits a high variability of activation motifs especially in early divergent plant species. (TIF) [file pone.0250584.s002.tif]

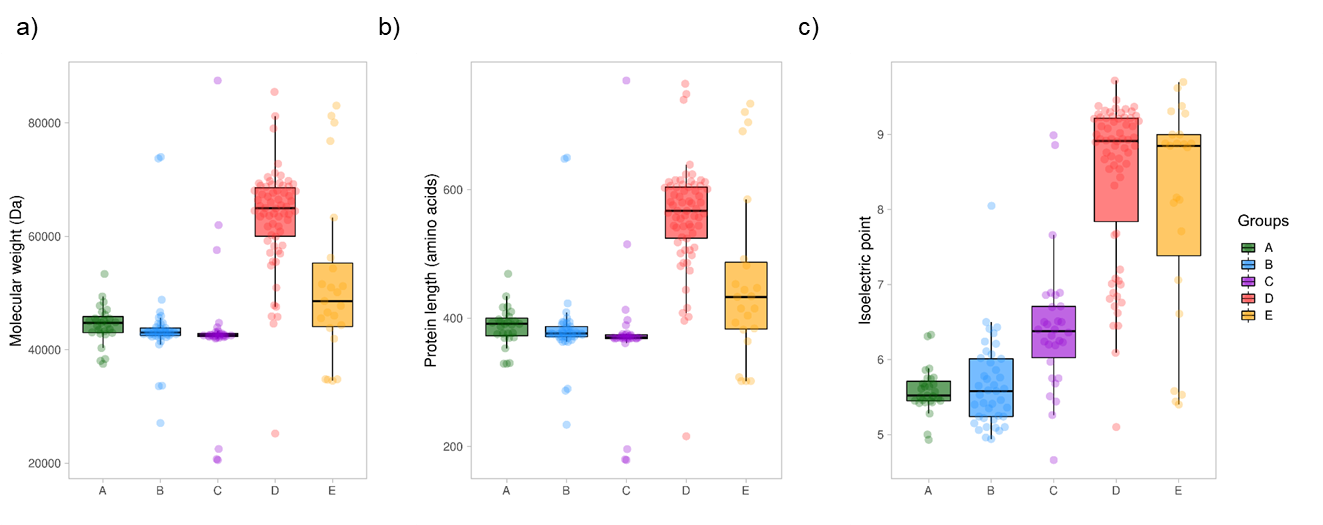

Supplement: S3 Fig — Boxplots showing a) molecular weight, b) protein length, and c) theorical isoelectric point of MAPK proteins according to their group. The boxplot indicates the median line as well as first and third quartiles. Outliers that are 1.5 above the upper quartile or below the lower quartile are indicated as points. (TIF) [file pone.0250584.s003.tif]

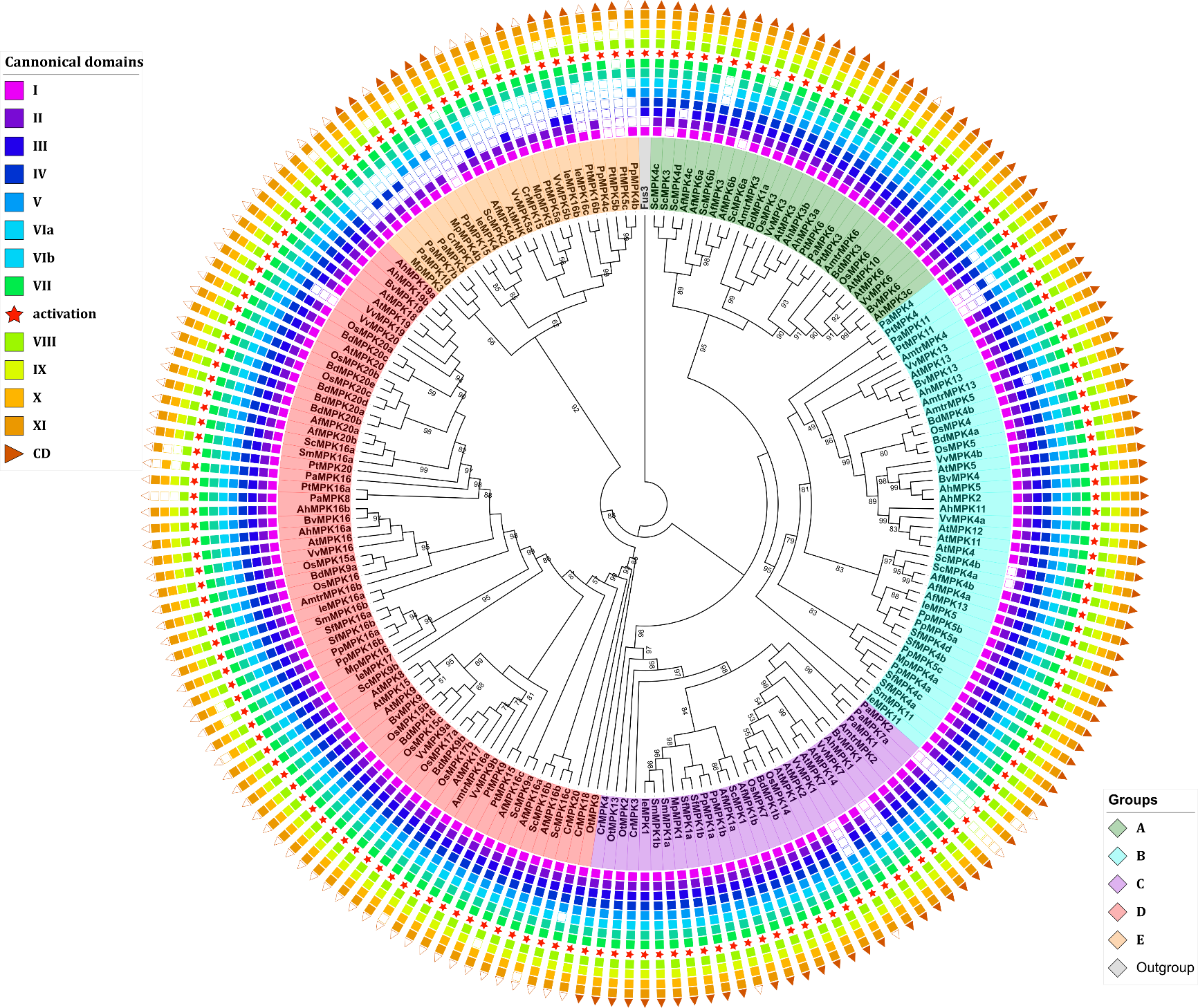

Supplement: S4 Fig — The identification of the canonical MAPK domains was performed with the MEME suite [44]. Sequence nomenclature follows a two-letter code to indicate genus and species (see text). (TIF) [file pone.0250584.s004.tif]

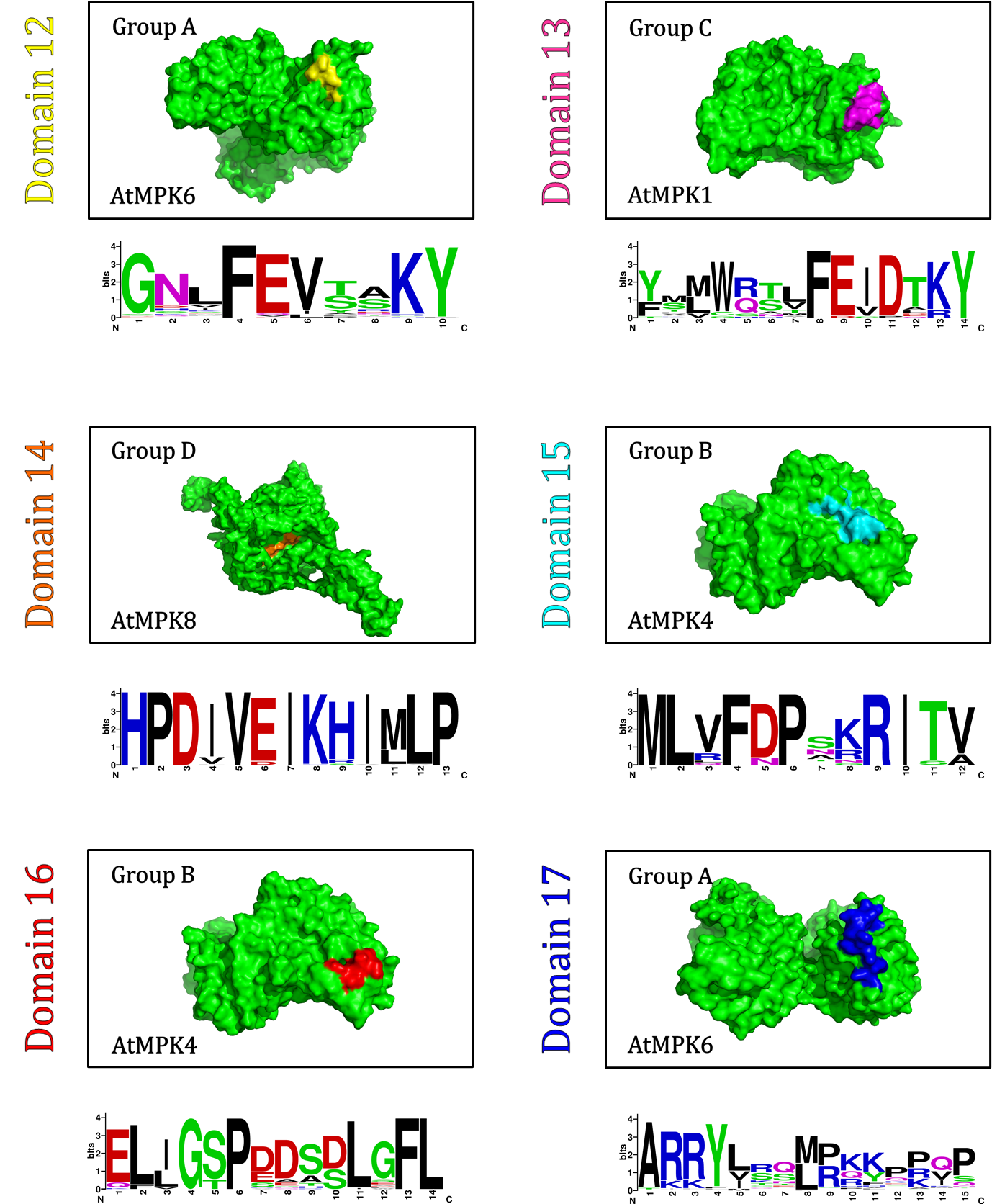

Supplement: S5 Fig — The protein structures were obtained from homology modelling [47] and visualized in PyMol (The PyMOL Molecular Graphics System, Version 2.0 Schrödinger, LLC.). Each domain is presented as a sequence logo, obtained from the multiple sequence alignment of all the retrieved sequences [46], and highlighted in the protein struure with a distinctive color. (TIF) [file pone.0250584.s005.tif]

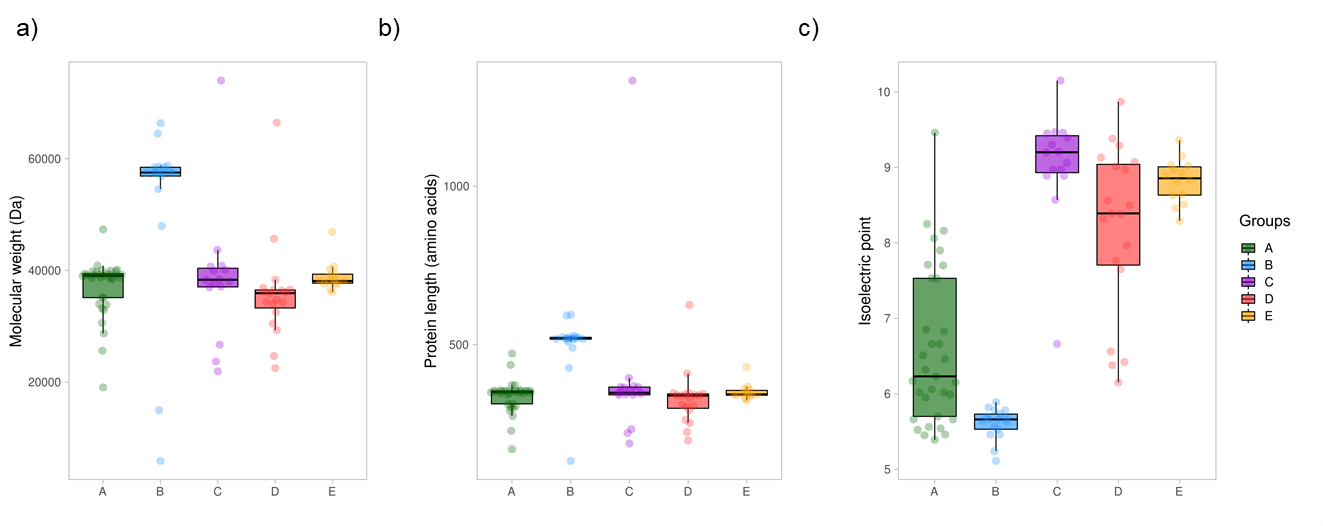

Supplement: S6 Fig — Boxplots showing a) molecular weight, b) protein length, and c) theorical isoelectric point of MEK proteins according to their group. The boxplot indicates the median line, first, and third quartiles. Outliers that are 1.5× above the upper quartile or below the lower quartile are indicated as points. (TIF) [file pone.0250584.s006.tif]

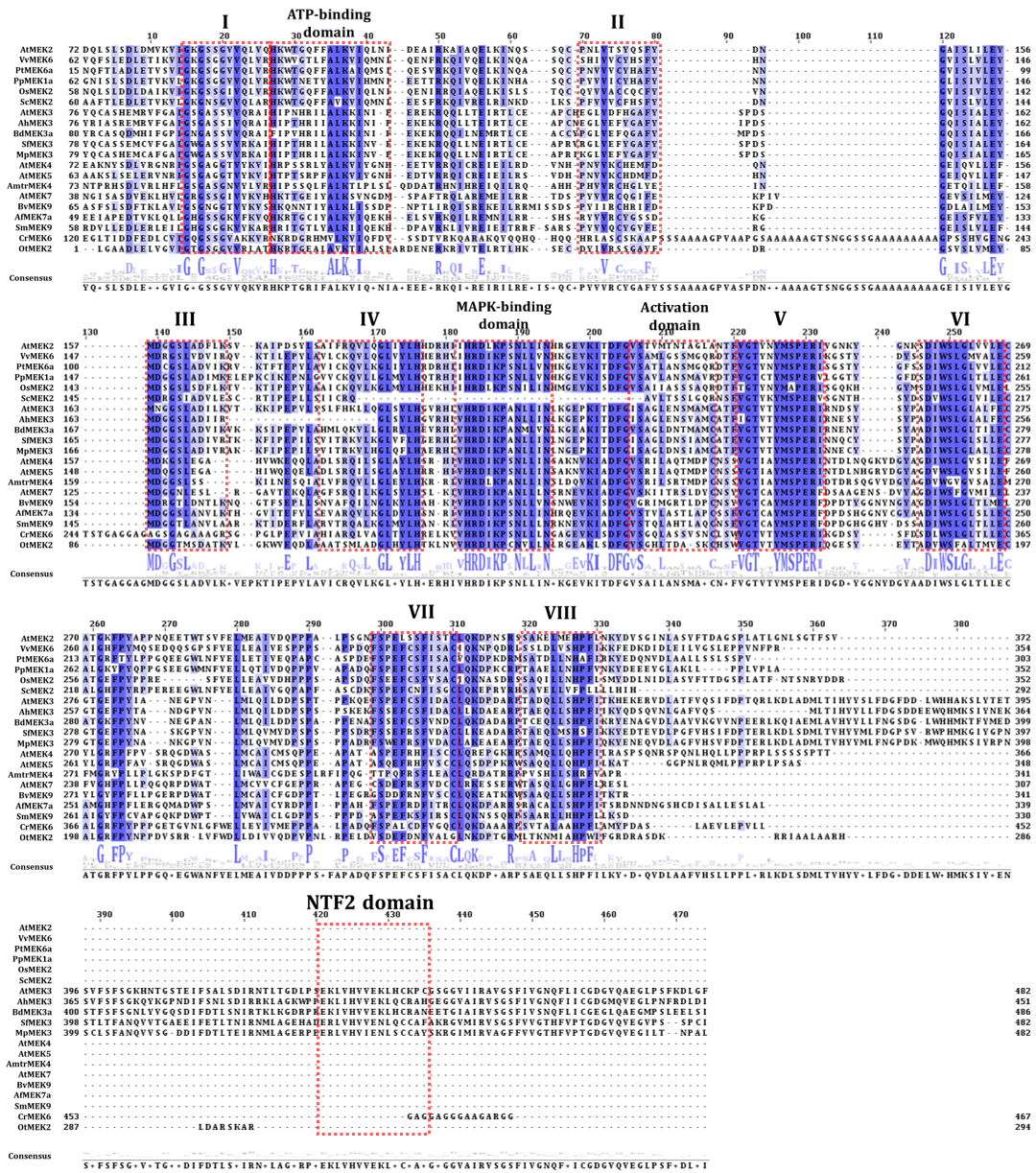

Supplement: S7 Fig — Twenty proteins representing the diversity of MEKs across Viridiplantae were aligned using the MAFFT software [39] and visualized in Jalview [40]. The numerals I to VIII represent the canonical domains; the ATP-binding and MAPK-binding domain are also indicated. (TIF) [file pone.0250584.s007.tif]

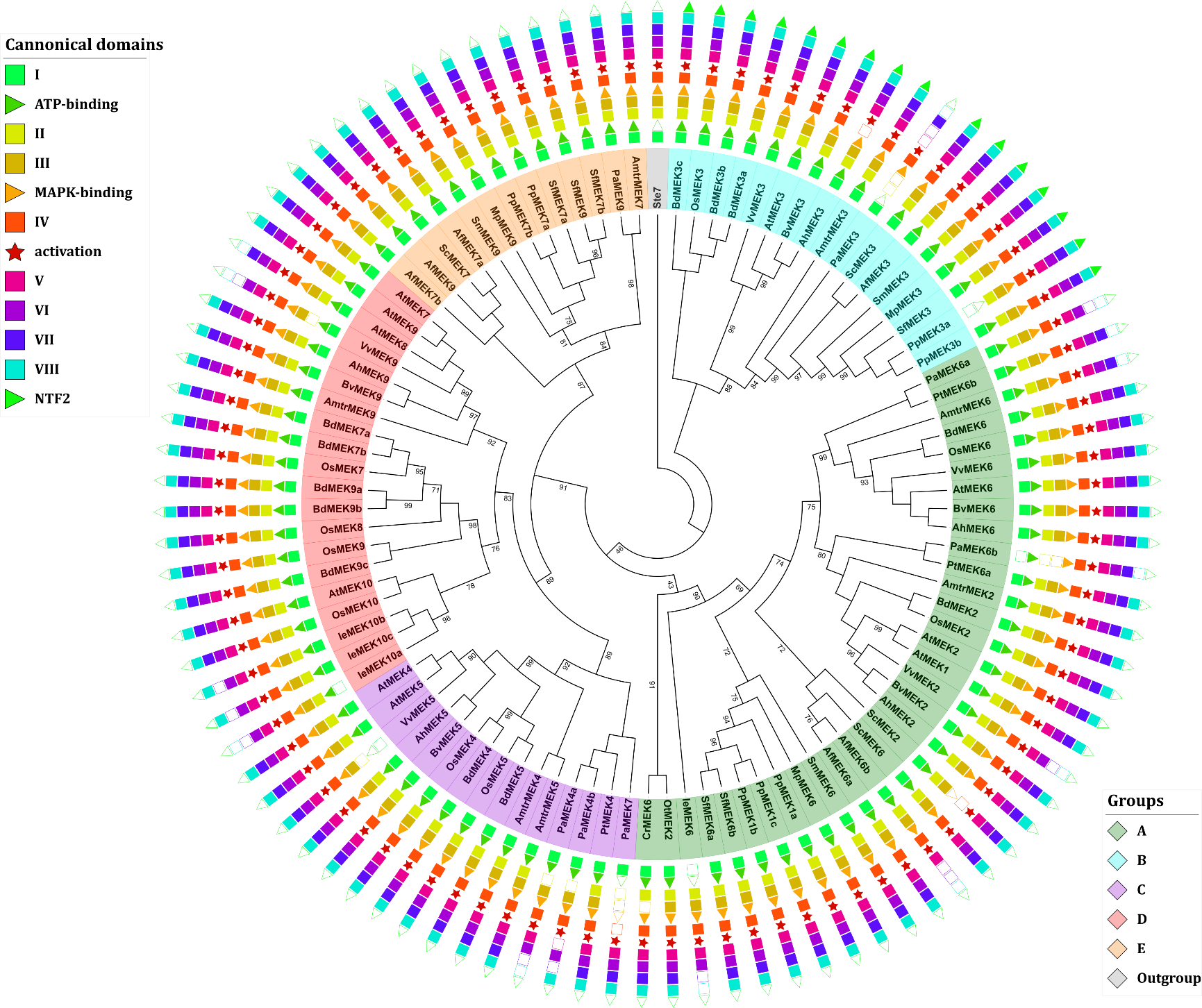

Supplement: S8 Fig — The identification of the canonical MEK domains was performed with the MEME suite [44]. Sequence nomenclature follows a two-letter code to indicate the genus and species (see text). (TIF) [file pone.0250584.s008.tif]
