# Supplementary material for: A phylogenetic study of the members of the MAPK and MEK families across Viridiplantae
Source: PLoS One. 2021 Apr 23;16(4):e0250584. doi: 10.1371/journal.pone.0250584 (PMC8064577; doi:10.1371/journal.pone.0250584)
Supplement: Attachment — Submitted filename: PONE-D-21-00271_reviewer-1_commented.pdf [file pone.0250584.s019.pdf]

PLOS ONE

Phylogenetic study of the members of the MAPK and. MEK families across Viridiplanta

--Manuscript Draft--

**Manuscript Number:**	PONE-D-21-00271
**Article Type:**	Research Article
**Full Title:**	Phylogenetic study of the members of the MAPK and. MEK families across Viridiplanta
**Short Title:**	Plant MAPKs and MEKs phylogeny
**Corresponding Author:**	Angel A. Guevara-García<br>Instituto de Biotecnología UNAM<br>Cuernavaca , Morelos MEXICO
**Keywords:**	Viridiplantae; Phylogeny; MEK gene family; MAPK gene family; Novel domains.
**Abstract:**	Protein phosphorylation processes are regulated by the activity of enzymes generically known as kinases. One of that kinases are the called Mitogen-Activated Protein Kinases (MAPK), which operate through a phosphorylation cascade conformed by members from three related protein kinase families, namely MAPK kinase kinase (MEKK), MAPK kinase (MEK) and MAPK, which act hierarchically. MAPK´s families are ubiquitous in eukaryotes and each one is constituted by a variable number of proteins in different species. In the Viridiplantae lineage, they have been implicated in the control of multiple processes, including stress responses and developmental programs. Establishing the evolution of these proteins in the vegetal kingdom is an interesting but very complicated task, as the currently existing MAPK, MAPKK, and MAPKKK subfamilies resulted from duplications and subsequent sub-functionalization during the early stage of the emergence of the Viridiplantae clade. Here, an in silico genomic analysis performed on 18 different plant species, that resulted in the identification of 96 genes not previously annotated as components of the MAPK (70) and MEK (26) families, is presented. Interestingly, a deeper analysis of the sequences encoded by such genes revealed the existence of putative domains not previously described as signatures of MAPK and MEK kinases. Additionally, our analysis also suggests the presence of conserved activation motifs besides the canonical TEY and TDY domains, which characterize the MAPK family.
**Order of Authors:**	José Manuel González-Coronel
	Gustavo Rodríguez-Alonso
	Angel A. Guevara-García
**Additional Information:**	
**Question**	**Response**
**Financial Disclosure**<br><br>Enter a financial disclosure statement that describes the sources of funding for the work included in this submission. Review the submission guidelines for detailed requirements. View published research articles from *PLOS ONE* for specific examples.<br><br>This statement is required for submission and **will appear in the published article** if the submission is accepted. Please make sure it is accurate.	The authors thank Gustavo Delgado-Prudencio and Adrian Martínez-Santana (graduate students in the Instituto de Biotecnología-UNAM) for their insightful suggestions and help with phylogenetic analysis. The technical assistance from MTI Juan Manuel Hurtado-Ramírez and MEM David Santiago Castañeda Carreón (Unidad de Cómputo del Instituto de Biotecnología-UNAM) is also greatly acknowledged. This work was supported by UNAM-DGAPA-PAPIIT (grant IN209420 to AAGG), PRODEP (postdoctoral fellowship granted to GRA), and CONACYT-México (fellowship 481085 to JMGC).<br>The funders had no role in study design, data collection and analysis, decision to publish, or preparation of the manuscript.

Powered by Editorial Manager® and ProduXion Manager® from Aries Systems Corporation

**Unfunded studies**<br>Enter: *The author(s) received no specific funding for this work.*<br><br>**Funded studies**<br>Enter a statement with the following details:<br>• Initials of the authors who received each award<br>• Grant numbers awarded to each author<br>• The full name of each funder<br>• URL of each funder website<br>• Did the sponsors or funders play any role in the study design, data collection and analysis, decision to publish, or preparation of the manuscript?<br>• **NO** - Include this sentence at the end of your statement: *The funders had no role in study design, data collection and analysis, decision to publish, or preparation of the manuscript.*<br>• **YES** - Specify the role(s) played.<br><br>* typeset	
**Competing Interests**<br><br>Use the instructions below to enter a competing interest statement for this submission. On behalf of all authors, disclose any competing interests that could be perceived to bias this work—acknowledging all financial support and any other relevant financial or non-financial competing interests.<br><br>This statement **will appear in the published article** if the submission is accepted. Please make sure it is accurate. View published research articles from *PLOS ONE* for specific examples.	All the authors have declared that no competing interests exist.

**NO authors have competing interests**<br><br>Enter: *The authors have declared that no competing interests exist.*<br><br>**Authors with competing interests**<br><br>Enter competing interest details beginning with this statement:<br><br>*I have read the journal's policy and the authors of this manuscript have the following competing interests: [insert competing interests here]*<br><br>* typeset	
**Ethics Statement**<br><br>Enter an ethics statement for this submission. This statement is required if the study involved:<br><br>• Human participants<br>• Human specimens or tissue<br>• Vertebrate animals or cephalopods<br>• Vertebrate embryos or tissues<br>• Field research<br><br>Write "N/A" if the submission does not require an ethics statement.<br><br>General guidance is provided below. Consult the submission guidelines for detailed instructions. **Make sure that all information entered here is included in the Methods section of the manuscript.**	N/A

Powered by Editorial Manager® and ProduXion Manager® from Aries Systems Corporation

**Format for specific study types**<br><br>**Human Subject Research (involving human participants and/or tissue)**<br>• Give the name of the institutional review board or ethics committee that approved the study<br>• Include the approval number and/or a statement indicating approval of this research<br>• Indicate the form of consent obtained (written/oral) or the reason that consent was not obtained (e.g. the data were analyzed anonymously)<br><br>**Animal Research (involving vertebrate animals, embryos or tissues)**<br>• Provide the name of the Institutional Animal Care and Use Committee (IACUC) or other relevant ethics board that reviewed the study protocol, and indicate whether they approved this research or granted a formal waiver of ethical approval<br>• Include an approval number if one was obtained<br>• If the study involved *non-human primates*, add *additional details* about animal welfare and steps taken to ameliorate suffering<br>• If anesthesia, euthanasia, or any kind of animal sacrifice is part of the study, include briefly which substances and/or methods were applied<br><br>**Field Research**<br><br>Include the following details if this study involves the collection of plant, animal, or other materials from a natural setting:<br>• Field permit number<br>• Name of the institution or relevant body that granted permission	
**Data Availability**<br><br>Authors are required to make all data underlying the findings described fully available, without restriction, and from the time of publication. PLOS allows rare exceptions to address legal and ethical concerns. See the PLOS Data Policy and FAQ for detailed information.	Yes - all data are fully available without restriction

A Data Availability Statement describing where the data can be found is required at submission. Your answers to this question constitute the Data Availability Statement and **will be published in the article**, if accepted.<br><br>**Important:** Stating 'data available on request from the author' is not sufficient. If your data are only available upon request, select 'No' for the first question and explain your exceptional situation in the text box.<br><br>Do the authors confirm that all data underlying the findings described in their manuscript are fully available without restriction?	
**Describe where the data may be found in full sentences. If you are copying our sample text, replace any instances of XXX with the appropriate details.**<br><br>• If the data are **held or will be held in a public repository**, include URLs, accession numbers or DOIs. If this information will only be available after acceptance, indicate this by ticking the box below. For example: *All XXX files are available from the XXX database (accession number(s) XXX, XXX.)*.<br>• If the data are all contained **within the manuscript and/or Supporting Information files**, enter the following: *All relevant data are within the manuscript and its Supporting Information files.*<br>• If neither of these applies but you are able to provide **details of access elsewhere**, with or without limitations, please do so. For example:<br><br>*Data cannot be shared publicly because of [XXX]. Data are available from the XXX Institutional Data Access / Ethics Committee (contact via XXX) for researchers who meet the criteria for access to confidential data.*<br><br>*The data underlying the results presented in the study are available from (include the name of the third party*	All relevant data are within the manuscript and its Supporting Information files.

Powered by Editorial Manager® and ProduXion Manager® from Aries Systems Corporation

and contact information or URL).<br>• This text is appropriate if the data are owned by a third party and authors do not have permission to share the data.<br><br>* typeset	
Additional data availability information:	Tick here if the URLs/accession numbers/DOIs will be available only after acceptance of the manuscript for publication so that we can ensure their inclusion before publication.

Powered by Editorial Manager® and ProduXion Manager® from Aries Systems Corporation

Phylogenetic study of the members of the MAPK and MEK families across Viridiplantae.

José Manuel González-Coronel [1], Gustavo Rodríguez-Alonso [1,2], Ángel Arturo Guevara-García [1*]

[1] Departamento de Biología Molecular de Plantas. Instituto de Biotecnología, Universidad Nacional Autónoma de México, Cuernavaca, Morelos, México.

[2] Centro de Investigación en Dinámica Celular, Instituto de Investigación en Ciencias Básicas y Aplicadas. Universidad Autónoma del Estado de Morelos, México

[*] Corresponding autor. Tel: +52 (777) 329-1777 ext 38134; Fax: +52 (777) 313-9988.

E-mail adress: aguevara@ibt.unam.mx

Abstract

Protein phosphorylation processes are regulated by the activity of enzymes generically known as kinases. One of ~~that kinases are the~~ called Mitogen-Activated Protein Kinases (MAPK), which operate through a phosphorylation cascade conformed by members from three related protein kinase families, namely MAPK kinase kinase (MEKK), MAPK kinase (MEK) and MAPK, which act hierarchically. MAPK´s families are ubiquitous in eukaryotes and each one is constituted by a variable number of proteins in different species. In the Viridiplantae lineage, they have been implicated in the control of multiple processes, including stress responses and developmental programs. Establishing the evolution of these proteins in the vegetal kingdom is an interesting but very complicated task, as the currently existing MAPK, MAPKK, and MAPKKK subfamilies resulted from duplications and subsequent sub-functionalization during the early stage of the emergence of the Viridiplantae clade. Here, an *in silico* genomic analysis performed on 18 different plant species, that resulted in the identification of 96 genes not previously annotated as components of the MAPK (70) and MEK (26) families, is presented. Interestingly, a deeper analysis of the sequences encoded by such genes revealed the existence of putative domains not previously described as signatures of MAPK and MEK kinases. Additionally, our analysis also suggests the presence of conserved activation motifs besides the canonical TEY and TDY domains, which characterize the MAPK family.

Keywords: Viridiplantae, Phylogeny, MEK gene family, MAPK gene family, Novel domains.

Introduction

Plants have evolved diverse and complex response mechanisms to contend against constantly changing environmental conditions. The perception of these conditions, together with the subsequent transduction and amplification of the generated signals, triggers cellular responses crucial to achieve optimal growth and development. Post-translational modifications (PTMs) constitute a major regulatory mechanism of protein activity [1,2]. PTMs regulate the activation or inhibition of protein activity, change in subcellular localization, alter the stability of proteins, and promote or prevent trans-interactions [1,3]. PTMs reactions are catalyzed by a wide variety of enzymes that are usually

reversible [1,4,5]. Particularly, protein kinases regulate processes at the transcriptional, translational and post-translational level by catalyzing the addition of a phosphate group (PO_4^{3-}) from ATP to a substrate protein in specific amino acid residues, usually serine (S), threonine (T) or tyrosine [Y] [6–8]. Phosphorylation reactions depend on the catalytic activity of the enzyme and the affinity, or protein-protein interaction capability, to their target proteins [5,9,10].

Plant genomes have a large number of genes encoding protein kinases, which constitute a protein superfamily [11,12]. Compared to other eukaryotic genomes, plants contain a larger number of protein kinases; for example, the *Arabidopsis thaliana* L. genome, contains 1,200 kinase genes approximately, while the rice (*Oryza sativa* L.) genome contains ~1,400 kinase genes [13,14]. On the other hand, approximately 500 and 120 proteinase kinases are encoded in the *Homo sapiens* L. and *Saccharomyces cerevisiae* Meyen ex E.C. Hansen. genomes, respectively [15]. The MAPKs belong to the group of serine/threonine protein kinases that are responsible for transforming extracellular *stimuli* into a wide range of cellular responses [5,16].

MAPKs are among the oldest signal transduction pathways and in plants are involved in the regulation of various physiological processes such as the detection of hormones [16–19] and the response to different types of stress [5,20–23]. MAPK-mediated signaling cascades are highly conserved in all eukaryotic organisms and are made up of three different gene families, named MAPK or MPK, MAPK kinase (MKK or MEK), and MAPKK kinases (MPKKK or MEKK) [5,24–26]. The components of the MAPK cascades are sequentially activated by phosphorylation of amino acid residues in the activation domain of their substrates [8,25,27]. The first component of the signaling cascade, MEKK proteins, phosphorylates a pair of serine (S) and threonine (T) residues in the S/T-X_{3-5}-S/T domain of MEK proteins. Subsequently, MEKs activate MAPKs by phosphorylation of threonine (T) and tyrosine (Y) residues in the activation domain (T-X-Y) [17,26,28].

Various members of the MEK family can act as convergence points of a great variety of signals and stimuli, with an extraordinary high substrate specificity towards a MAPK. MAPKs, on the other hand, seem to function as divergence points [29]. It has been suggested that the modular and hierarchical arrangement of the MAPK signaling pathways is suitable for amplification and integration of various

signals at the cellular level [29,30]. Signaling cascades mediated by MAPKs coexist in many cells and are connected and regulated by feedback to at least some degree [1,16,19,24,29,31]. Due to the central role played by the MAP kinases in signal transduction and transmission, it is of great interest to classify the members of these families based on their phylogenetic relationships and their functional characteristics [29,32].

The availability and analyses of plant genome sequences have facilitated the identification of various members of the MAP kinase family in numerous plant species, contributing to their evolutionary study, as well as, the elucidation of the signaling mechanisms involved in various types of responses [18,20,28,33–36]. Despite the growing availability of plant genomes, the identification of MPKs has been limited to a small number of species, mainly from monocots [36–38], and dicots [17,18,20,26,27,33,39–43].

In *Arabidopsis thaliana* L. genome 20 genes encoding MAPKs, 10 for MEK and 80 for MEKK have been identified. In general, the number of genes that encode members of the MEK family is much smaller that of the MAPK and MEKK families [17,21,31,35]. MEKK proteins represent the largest family of signaling cascade components mediated by MAP kinases with 80 members classified into three groups: MAPKKK, ZIK and RAF, consisting of 21, 11 and 48 genes respectively [31]. Members of the MAP kinase family are characterized by having 11 canonical domains, with an activation domain between domains VII and VIII. The activation domain of MAP kinases contains a pair of threonine and tyrosine residues, which are phosphorylated by a MEK protein [44]. Most of the angiosperm MAPKs have been classified into 4 groups (A-D) on the basis of their activation domain [17].

In *Arabidopsis*, the participation of various MAP kinases modules in response to different stimuli has been evidenced, such as the MEKK1-MKK4/5-MPK3/6 module, which participates in the perception and signaling of the flagellin-mediated immune response [45]; the MEKK1-MEK1/2-MPK4/6 module, which is activated in response to different types of stress [46]; and the module MEKKK4-MEK4/5-MPK3/6, initially identified as a regulator of stomata development [47], and later suggested to participate in the regulation of root and embryo development and morphology [25]. In this work, we

identified MAP kinase genes of seven species not previously analyzed: *Amaranthus hypochondriacus* L., *Azolla filiculoides* [Lam.], *Marchantia polymorpha* L., *Pinus taeda* L., *Ostreococcus tauri* C. Courties & M.-J. Chrétiennot-Dinet, *Salvinia cucullata* [Bory.], and *Isoetes echinospora* Durieu.; and provide a list of members of the MEK and MAPK families of each of the eighteen analyzed species. So, a total of 70 and 26 novel sequences of MAPK and MEK proteins were obtained, respectively. In addition to a detailed study of the members of the MAP kinase family of the aforementioned species, a comparative analysis was carried out by adding the genes that code for the members such families in representative species of the different plant lineage, including a chlorophyta (*Chlamydomonas reinhardtii* PA Dang.), a lycophyte (*Selaginella moellendorffii* P. Beauv.), two bryophytes (*Physcomitrella patens* [Hedw.] Bruch & Schimp., and *Sphagnum fallax* H. Klinggr.), a gymnosperm (*Picea abies* [L.] H. Karst.), and four angiosperm species: *Vitis vinifera* L., *Beta vulgaris* L., *Brachypodium distachyon* [L.] P. Beauv. and *Amborella trichopoda* Baill.

Materials and Methods

Identification of MAPK and MEK genes in Viridiplantae

A BLASTP was carried using the sequences of the MAPK and MEK proteins of *Arabidopsis thaliana* L. as a query to find putative orthologs in *Amaranthus hypochondriacus* L., *Amborella trichopoda* Baill., *Chlamydomonas reindhardtii* P.A. Daung., *Physcomitrella patens* [Hedw.] Bruch & Schimp., *Selaginella moellendorffii* P. Beauv., *Sphagnum fallax* H. Klinggr. (https://phytozome.jgi.doe.gov/pz/portal.html), *Azolla filiculoides* [Lam.], and *Salvinia cucullata* [Bory.] (https://www.fernbase.org/), *Marchantia polymorpha* L. (https://marchantia.info), *Oryza sativa* L. (http://www.plantgdb.org/OsGDB/), *Ostreococcus tauri* C. Courties & M.-J. Chrétiennot-Dinet. (https://genome.jgi.doe.gov/Ostta4/Ostta4.home.html), *Pinus taeda* L., *Picea abies* [L.] H. Karst. (http://congenie.org/), and *Vitis vinífera* L. (http://www.genoscope.cns.fr/externe/GenomeBrowser/Vitis/) databases; considering an expect value (e-value) of $1x10^{-35}$ as a threshold. An additional search was carried out using Hidden Markov

Models using the HMMER 3.0 software [48]. Subsequently, the presence of the serine / threonine protein kinase domain (PF00069) was corroborated in the, retrieved sequences.

Those proteins containing the D-[L/I/V]-K and S/T-X_{3-5}-S/T sequences were considered putative MEK proteins (17,37). Whereas, the putative members of the MAPK family must contain the characteristic sequence [L/I/V/M]-[TS]-X-X-[L/I/V/M]-X-T-[K/R]-[W/Y]-Y-R-X-P-X-[L/I/V/M]-[L/I/V/M], including the T-X-Y activation domain [17,18]. The NCBI Conserved Domain Database (http://blast.ncbi.nlm.nih.go), ProtParam online software from the ExPASy suite (https://web.expasy.org/protparam/), and the InterProScan database (https://www.ebi.ac.uk/interpro/search/sequence-search) were used to validate the presence of domains in each of the sequences. Finally, the molecular weight (MW) and the isoelectric point (pI) of each protein was predicted with the ExPASy server (http://web.expasy.org/compute_pi/). Boxplots for molecular weight and isoelectric point were visualized using PlotsOfData software (https://huygens.science.uva.nl/PlotsOfData/).

Multiple sequence alignment, phylogenetic, and gene structure analysis

Multiple sequence alignment was generated using the iterative refined method E-INS-i from MAFFT online software (Multiple Alignment using Fast Fourier Transform; https://mafft.cbrc.jp/alignment/server/) [49,50], (S1 and S2 Files) and visualized with Jalview software v2.11.1.3 [51]. The IQtree 1.6.6 (http://www.iqtree.org/) and ProtTest (http://darwin.uvigo.es) software were used to determine the evolution substitution model of proteins to be used for the construction of the phylogenetic tree [52]. The phylogenetic analysis was performed using the complete sequence of each protein with the IQtree v1.6.6 software [53]. The Maximum-Likelihood algorithm was used. The tree topology was statistically tested with the bootstraps method with 1,000 iterations (S3 and S4 Files). The tree topology was visualized in the iTOL software v5.7 [54].

Identification of novel domains and protein structure prediction

To identify the canonical and novel MAPK and MEK domain, the MEME-suite software v5.2.0 [55] was used. MEME was run with default parameters and predefined motif length of eight to 12-15 residues. The PhosphoSVM software [56] was used to predict phosphorylation of the novel domains. The protein logos was performed with WebLogo software v2.8.2 [57].

The protein structure was predicted with the I-Tasser software v5.1 [58] and visualized in the PyMol software v2.4.1 (The PyMOL Molecular Graphics System, Version 2.0 Schrödinger, LLC.), MPK6 crystal structure (PDB id: 5ci6) [59] was used as template for structure modelling. Figure composition was performed using the InkScape software v1.0 (https://inkscape.org/es/).

Results and Discussion

Identification and nomenclature

MAP kinases are of paramount importance for plant development and stress response [1]. The partial redundancy of this protein superfamily, the high sequence similarity among homologs, and the expansion of the gene family in diverse plant species, has hindered the evolutionary studies of MAPKs. To provide a comprehensive phylogenetic analysis of MAPK proteins, a genome-wide identification of MAPK genes was performed for 18 selected species, spanning the major clades of Viridiplantae. Our analysis includes chlorophyta (*C. reindhardtii*, and *O. tauri*), bryophytes (*M. polymoprpha*, *P. patens*, and *S. fallax*), lycophyte (*S. moellendorffii*), seedless vascular plants (*A. filiculoides*, *I. echinospora*, and *S. cucullata*), gymnosperms (*P. taeda* and *P. abies*) and angiosperms. For the last clade we include the basal angiosperm *Amborella trichopoda*, as well as monocotyledonous (*B. dystachion*, and *O. sativa*) and dicotyledoneous species (*A. hypochondriacus*, *A. thaliana*, *B. vulgaris*, and *V. vinifera*). MAPK genes were identified by a combined approach of homology identification and hidden Markov models (Table 1).

Table 1. MAPK genes presents in different plant genomes.

	Specie	Lineage	MAPKs gene number
1	*Arabidopsis thaliana* L.	Angiosperm (Eudicot)	20
2	*Amaranthus hypochondriacus* L.	Angiosperm (Eudicot)	12
3	*Beta vulgaris* L.	Angiosperm (Eudicot)	7
4	*Vitis vinifera* L.	Angiosperm (Eudicot)	14
5	*Brachypodium distachyon* [L.] P. Beauv.	Angiosperm (Monocot)	14
6	*Oryza sativa* L.	Angiosperm (Monocot)	15
7	*Amborella trichopoda* Baill.	Angiosperm (Basal)	8
8	*Picea abies* [L.] H. Karst.	Gymnosperm	11
9	*Pinus taeda* L.	Gymnosperm	12
10	*Azolla filiculoides* [Lam.]	Pteridophyta	15
11	*Salvinia cucullata* [Bory.]	Pteridophyta	14
12	*Isoetes echinospora* Durieu.	Lycophyta	8
13	*Selaginella moellendorffii*	Lycophyta	5
14	*Sphagnum fallax* H. Klinggr.	Bryophyta	8
15	*Marchantia polymorpha* L.	Bryophyta	6
16	*Physcomitrella patens* [Hedw.] Bruch & Schimp.	Bryophyta	11
17	*Chlamydomonas reindhardtii* P. A. Dang.	Algae	6
18	*Ostreococcus tauri* C. Courties & M.-J. Chrétiennot-Dinet	Algae	3

This analysis resulted in the identification of 153 annotated MAPK genes and 96 genes not previously annotated as members of any of the three different modules that constitute a canonical MAP kinase cascade (S1 Table). 70 of the novel MAPK genes belong to MAPK and 27 to the MEK subfamily, respectively. Although previous efforts to reconstruct the evolutionary history of MAPK genes had been made, ortholog identification on early divergent plant clades has remained elusive [27].

Moreover, independent studies in species such as *A. thaliana*, *O. sativa*, and *P. trichocarpa*, has led to different nomenclature and classification systems for MAPKs [17].

For our part, taking the MAPKs as an example for the establishment of a nomenclature, each of the genes was named using a two letters code corresponding to the first letter from the genus and species. This two-letter code remained in: Af (*Azolla filiculoides*), Ah (*Amaranthus hypochondriacus*), At (*Arabidopsis thaliana*), Bv (*Beta vulgaris*), Bd (*Brachypodium distachyon*), Cr (*Chlamydomonas reindhardtii*), Ie (*Isoetes echinospora*), Mp (*Marchantia polymorpha*), Ot (*Ostreoccocus tauri*), Os (*Oryza sativa*), Pp (*Physcomitrella patens*), Pa (*Picea abies*), Pt (*Pinus taeda*), Sc (*Salvinia cucullata*), Sf (*Sphagnum fallax*), Sm (*Selaginella moellendorffii*), and Vv (*Vitis vinífera*); to distinguish *Amborella trichopoda* from *A. thaliana*, the second letter of both, genus and species, species was added (i.e Amtr). Next to this letter code the acronym MPK (from **M**itogen-activated **P**rotein **K**inase) or MEK (MAPK/ERK Kinase) was included, and a number referring to its most likely ortholog in *Arabidopsis thaliana* L [27]. Likewise, when two or more of the identified sequences have the same putative ortholog in *Arabidopsis*, they were distinguished by adding a letter in alphabetical order. Such is the case of the *P. taeda* sequences PITA_000030510, PITA_000007088 and PITA_000001460, which, being putative orthologs of AtMPK5, were renamed as PtMPK5a, PtMPK5b and PtMPK5c, respectively.

Phylogenetic analysis of MAP Kinases

A multiple sequence alignment of the retrieved MAP Kinases sequences was built and used to reconstruct the molecular phylogeny of MAP kinases. The alignment evidenced that there is a high degree of conservation in the sequences that correspond to the 11 characteristic MAPK domains; moreover, all analyzed sequences contain the TXY- activation domain (S1 Fig). A Maximum Likelihood tree was constructed for the MAP Kinase retrieved sequences; the tree was rooted by using the *Saccharomyces cerevisiae* Fus3 protein, which was selected as an outgroup given its similarity to Viridiplantae MAPKs. Previous studies classified the *Arabidopsis* MAPK genes into four groups (A, B, C and D), according to their sequence similarity and the presence the TDY or TEY phosphorylation motifs [17]. The obtained ML tree topology displays five well supported clades, with

bootstrapping values >90.0% out of 1000 replicates, therefore we support the previous suggestion to consider another group to classify MAP kinases, named A-E, but at the same time we refute the existence of an additional group (F) [34] (Fig 1).

Fig 1. Phylogenetic analysis of Viridiplantae MAPKs proteins. Maximum-likelidood tree reconstructed from amino acid sequences. The tree was rooted with the Fus3 protein from Saccharomyces cerevisiae. Bootstrap values from 1,000 replicates are indicated at nodes with support values >49%. Each MAPK group is indicated with a distinctive color. Different species are indicated with shapes and colors.

The MAPK group A contains sequences retrieved from pteridophytes, gymnosperms and angiosperms. Surprisingly, no group A MAPKs were identified in the lycophytes, bryophytes nor algae species analyzed. This observation suggests that group A MAPKs arose in the Euphyllophyte clade, after the separation from the lycophytes, which occurred ca. 420 million years ago (MYA) [60,61] (Fig 1 and 2; S1 Table). AtMPK3 and AtMPK6, both belonging to the group A MAPKs, are the two most widely studied MAPK proteins, and together with their putative orthologs in other plant species, have been involved in responses to biotic and abiotic stresses [31,62,63]. Members of group B have been involved in cell division and in the responses to biotic and abiotic stress; in particular, it has been shown that the loss of function mutant *mpk4* of *A. thaliana* leads to a constitutive phenotype of systemic acquired resistance [64,65]. It has also been shown that some members, such as AtMPK13, another group B MAPK, are activated through the cell cycle and they are located specifically in the phragmoplast during telophase[66].

Fig 2. MAPKs abundances in Viridiplantae. a) Presence of the MAPK groups across Viridiplantae species. **b)** Number of MAPKs in each group in each species analyzed. Group E MAPKs, including AtMHK (●) were considered in the analysis although its recognition as a MAPK subfamily is still debated (see text). Colors in b) refer to the color used in Fig 1.

According to the obtained tree topology, groups A and B are sister clades; this observation, together with its absence in algae species, suggests that these MAPK groups were the last to be acquired (Figs 1 and 2).Group C MAPKs are less characterized than MAPKs from groups A and B, although it

has been shown that the expression and activity of AtMPK7, which belongs to the group C, is regulated by the circadian cycle [67]. Group D makes up the largest group of MAPK proteins, including 61 sequences, and it is characterized by the sequence TDY in its activation domain (S2 Fig); in addition, group D MAPKs have an extended C-end compared to proteins from the groups A-C. In *Arabidopsis*, the AtMPK8 protein from group D is involved in seed germination and dormancy [68].

Finally, members of group E are known as **M**apk-**H**omologous **K**inases (MHKs) and may contain the TEY or TDY sequence and even some non-canonical motif in the activation domain (S2 Fig) [17]. It has been a matter of debate whether group E MAPKs constitute functional MAPKs given its similarity to cyclin-dependent kinases (CDKs), even though their biochemical properties are largely unknown.

MAPK Analysis

Of the genomes under study, the angiosperm clade has the highest number of MAPK members per family, with *A. thaliana* being the species that contains the highest number of genes encoding MAPKs [20], followed by rice (*O. sativa*) and grape (*Vitis vinifera*) with 15 and 14 genes, respectively. In addition to this, in pteridophytes clade, the species *A. filiculoides* and *S. cucullata* have 15 and 14 genes, respectively. The large number of MAPK genes in Azolla and Salvinia might be a result of whole genome duplication events in the Salviniales [69]. On the other hand, in the algae group, *O. tauri* has the lowest number of genes of the analyzed species (3), while *C. reindhardtti* has 6. In bryophytes, there is an increase in the number of MAPK genes with respect to that of algae, the members of this clade, *P. patens*, *S. fallax*, and *M. polymorpha* contain 11, 8, and 6 genes, respectively. MAPK gene family expansion in bryophytes is congruent with the expansion of gene families in land plants upon terrestrialization and might be associates with plant facing new types of stress, such as dehydration, gravity, exposure to ultraviolet rays, etc. [70] after land colonization [71,72] (Fig 1; Table 1).

Regarding their size, among the 162 MAPK proteins identified, those belonging to group D have the longest length, being VvMPK7 (GSVIVT01018883001) of *V. vinifera* the longest protein with a size of 770 amino acids. In general, MAPKs proteins that belong to organisms from higher taxa such as angiosperms and gymnosperms have longer sequence than organisms of lower taxa, an exception

being CrMPK18 (Cre17.g745447) of the alga *C. reinhardtii* with a length of 765 amino acids. On the other hand, the PaMPK2 protein from *Picea abies* has the shortest length (172 amino acids). Regarding the structure of the MAPK gene, there seems to be no specific pattern of intron organization in terms of the taxonomic level to which each species belongs, finding genes with zero (6 sequences), one (18), two (8), three (5), four (5), five (46), six (8), seven (10), eight (8), nine (33), ten (20), eleven (2), twelve (2), fourteen (2), fifteen (7) and sixteen (1) introns. The MpMPK3 gene of *M. polymorpha* contains the maximum number of introns (16), while none of the three genes identified in the unicellular algae *O. tauri* have introns. Also, 18 of the sequences listed above contain at least one intron in the 3' or 5' UTR. (**U**n**T**ranslated **R**egion) according to their annotated gene models (S1 Table). However, regarding the organization of the groups, it is observed that those proteins that belong to groups D and E have the highest number of introns, while the members of group C present the least amount. Notably, gymnosperm and angiosperms organisms analyzed contain at least one intron. The predicted molecular weight of each of the MAP kinases reported here range from 20,559 Da (PaMPK2) to 87,480 Da (VvMPK7); while their isoelectric points (Ip) range from 4.66 (PaMPK2) to 9.72 (AfMPK16a) (S1 Table). Interestingly, group D and E comprise the largest proteins, with and average peptide length of 558 and 461, respectively (63,358 and 52,287 Da); these groups contain the most basic theorical isoelectric points, averaging 8.45 and 8.10, respectively (S3 Fig).

Analysis of motifs and domains conserved in the MAPK family

MAPKs are characterized by the presence of multiple domains. Eight domains, named I-V, VIa, VIb, and VII, are distributed towards the N-terminal, preceding the activation domain. While, towards the C-terminal end of the activation domain, there are five other domains previously described as typical of MAPKs. Domain VIII, IX, and X precede the CD domain; finally, around 30 amino acids after the CD domain, the XI domain is localized (Table 2) [44,46]. To address domain conservation among the identified MAPK sequences, we used the MEME's bioinformatics software [55].

Table 2. Consensus sequence of characteristic MAP kinase domains.

Domain	Consensus sequence
I	[V/P]-[I/V]-G-[K/R]-G-[S/A]-Y-G-[V/I]-V-C-S-A

II	E-X-V-A-I-K-K-I-X-[N/D]-[A/V/I]-F-[E/D]-[N/H]-X_2-D-A
III	R-[T/I]-L-R-E-[I/L]-K-L-L-R-[H/L]-[L/M]-[R/D]
IV	P-X-[R/K]-X_2-F-X-D-[V/I]-Y
V	V-[F/Y]-E-L-M-[E/D]-[T/S]-D-L-H-Q-[V/I]-I-[K/R]
VIa	[F/Y]-F-L-Y-Q-[L/I/M]-L-R-[G/A]-L-K-Y
VIb	H-[S/T]-A-N-[V/I]-[L/F/Y]-H-R-D-K-L-P-[K/S]-N-[L/I]-L-[A/L]-N
VII	C-[D/K]-L-K-I-[C/A]-D-F-G-L-A-R-[V/T]
VIII	[V/A]-T-R-W-Y-R-A-P-E-L-[L/C]-[L/G]-[S/N]
IX	A-I-D-[I/V/M]-W-S-[V/I]-G-C-I-F-[A/M]-E-[L/I/M]-[L/M]
X	P-[L/I]-F-P-G-X_3-[V/L]-X-Q-L-X-L-[I/M]-T-[D/E]
XI	F-D-P-X_2-R-[I/P]-[T/S]-[A/V]-X-[E/D]-A-L-X-[H/D]-P-Y-[F/L]
CD	L-H-D-X_2-D-E-P

Of the 90 sequences analyzed that belong to angiosperms, 83 present all the canonical domains. While the remaining seven (2 sequences from *A. trichopoda* and 5 from *V. vinifera*) lack at least one domain: VvMPK1 and VvMPK7 (domains I-IV), VvMK5a and VvMPK5b (III-V and VIa), VvMPK20 (I -III), AmtrMPK5 (IV), and AmtrMPK16a (XI). The fact that the vast majority of sequences belonging to angiosperms contain all the canonical domains could reflect a bias, since this clade is the most studied (Fig 3; S2 Table). Most of the sequences of the analyzed angiosperm species lack multiple domains. In *P. taeda*: PtMPK5a (III-V, and VIa), PtMPK5b (III-V, VIa, and IX), PtMPK5c (III-V, VIa, and IX-X), PtMPK11 (I -III), PtMPK16 (II-V, and VIa), PtMPK20 (IX-X). While, in *P. abies*: PaMPK2 (I-VII), PaMPK4a (V), PaMPK7a (VIb and VII-XI), PaMPK7b and PaMPK10 (III, V, and VIa), PaMPK11 (I-III, and V), and PaMPK16 (IX and XI). Eight of this sequences belong to group E MAPKs, which include MHKs and whose inclusion as MAPKs, as already mentioned, has been debated due to their sequence similarity to CDKs [24]. Whether group E proteins function as MAPKs has been poorly studied, although an ortholog of these proteins in the fungus *Ustilago maydis* is activated by phosphorylation by a MAPKK and contains the activation motif TXY [73]. This observation supports the hypothesis that group E MAPKs should be regarded as a subfamily of MAPKs. In fern species, the AfMPK6b and AfMPK16b sequences of *A. filiculoides* lack domain I and domain IX, respectively. While the AfMPK4d sequence lacks domains IV and IX. On the other hand, in *S. cucullata*, the ScMPK6b sequence lacks the V, VIa and VIb domains; the ScMPK16c sequence lacks domain XI.

Domains III, IV and VII are absent in the ScMPK2 sequence. Finally, domain I is absent in the ScMPK3, ScMPK4a and ScMPK4b sequences. In the case of *I. echinospora*, the sequence IeMPK4 lacks I-V domains, while IeMPK7 and IeMPK11 lacks domain XI (Fig 3; S2 Table).

Fig 3. Canonical domains in Viridiplantae MAPK proteins. The identification of the canonical MAPK domains was performed with the MEME suite [55]. Sequence nomenclature follows a two-letter code to indicate genus and species (see text).

The lack of several domains in the angiosperm and fern sequences is due to the fact that, currently in the databases, the genomes of these species are still fragmented, and possibly some of the ORFs that are found as hits comes from fragmented sequences and therefore the sequences corresponding to certain domains are missing. In the bryophytes *M. polymorpha*, *S. fallax,* and *P. patens*; MpMPK3 and MpMPK5 (III-V, and VIa), MpMPK4b (III and IV), PpMPK4b (II-IV, VIa, and IX), PpMPK4c (III-V, VIa, and IX), PpMPK15 (III, IV, VIa, and IX), and PpMPK16a y PpMPK16b(IX). For their part, the three sequences identified here from the alga *O. tauri* lack domain IV. Thus, the results of this analysis allow us to suggest that the presence of the activation motif (T-X-Y), together with five domains (II, III, V, and VII), located towards the N-terminal end of the activation domain and present in all the analyzed sequences, are enough to identify MAP kinases from any plant species. In addition to this, the presence of domains VIII-XI in most Viridiplantae MAPK sequences suggests that these domains are of critical importance for maintaining either the function or structure of these proteins (Fig 3; S2 Table).

MAPK proteins contain a T-X-Y motif, whose phosphorylation leads to MAPK activation [17]. The Viridiplantae multiple sequence alignment shows that all analyzed species contain the canonical activation domain in the form of T-E-Y (98 sequences), T-D-Y (77 sequences), and T-S-Y (1 sequence). The alignment also showed the presence of non-canonical activation domains, including M-E-Y (2 sequences), T-E-M (2 sequences), T-H-E (4 sequences), T-H-L (1 sequence), T-H-Q (1 sequence), T-K-T (2 sequences), T-Q-M (1 sequence), and T-S-Y (1 sequence). The distribution of these activation domains on the MAPK phylogeny shows that the T-E-Y domain is present in MAPKs from the groups A, B, C, and E; while the T-D-Y is present on the D and E groups (S2 Fig; S2 Table).

The activation domain contained in group E sequences exhibits the highest variability, which is especially evident on Bryophyta, including T-D-Y, T-H-E, T-K-T, and T-Q-M variants (S2 Fig).

In addition to the eleven characteristic domains, MAP kinases also contain the sequence (L/H)-D-X_2--D-E-P, known as the Common Docking domain (CD domain) [31]. Our results show that the CD domain is present in most proteins from the A, B, and C MAPK group; and although it is also present in a few sequences from the groups D and E, it is generally absent in proteins from these clades (Fig 3; S2 Table). The CD includes a motif involved in recognition and binding to MAPK substrates, as well as in protein-protein interaction with MEKs. The adjacent aspartate (D) and glutamate (E) residues in the CD, are essential for interaction with the basic residues lysine (K) and arginine (R), located in the binding site of MEK proteins [74]. Whether the absence of the CD domain has any impact on the function of these group D and E MAPKs, either for interaction and phosphorylation by MEKs or in MAPKs substrate specificity, remains to be experimentally demonstrated.

Identification of novel and distinctive domains for each group of MAPKs

The domain analysis of the retrieved sequences suggests the existence of six novel domains, named 12-17 (Fig 4; S3 Table). The presence of these domains might predict the group to which a MAPK protein belongs, facilitating MAPKs identification during genome or transcriptome annotation. The consensus sequences of such domains consist of 12-17 amino acid residues, and are reported in Table 3.

Fig 4. Identification of novel domains in Viridiplantae MAPK proteins. The analysis of novel domains was performed with the MEME suite [55]. The distribution of the novel domains is group specific and could, therefore, facilitate MAPK identification and annotation. Sequence nomenclature follows a two-letter code to indicate genus and species (see text).

Table 3. Consensus sequence of novel MAPKs domains

Domain	Consensus sequence
12	G-N-X-F-E-V-[T/S]-X-K-Y

13	Y-X-[M/L]-W-[Q/R]-[T/S]-X-F-E-I-D-T-K-Y
14	H-[P/K]-D-I-V-E-[I/V/K]-[K/L]-[I/H/N]-[I/K]-[M/L]-L-P
15	M-L-X-F-[D/N]-P-X_2-R-I-[T/S]
16	E-[L/V]-[I/L]-G-[T/S]-P-X-[E/D]-X-D-L-X-F-[L/I/V]
17	A-[R/K]-[R/K]-Y-[L/I/V]-X_2-[L/M]-[R/P]-X_3-[P/R/K]-X-[P/S]

Domain 12 is located approximately 15 residues before domain I from group A and B proteins. It is also present in OtMPK13, from *O. tauri*. Domain 13 is also located towards the N-terminus end of domain I but it is only present in the proteins from group C, therefore providing a signature for group C MAPKs. Domain 14 is located between domains II and III and typifies proteins from group D. Domains 15-17 are distributed towards the C-terminal end and, if present, they are always located before the CD domain, between the canonical domains X and XI. Domain 15 is present in proteins from the B and E group, with the sole exception of PtMPK16b from group E. With the exception of VvMPK3 and VvMPK6, which belong to group A, domain 16 is located only in group B MAPKs. Domain 17 is present in most proteins, except in those from groups C and E. Interestingly, domain 17 is also present in the Fus3 kinase of *S. cerevisiae*, which therefore prompts the hypothesis that this domain was present in the ancestral MAPK sequences and was lost in groups C and E (Fig 4; S3 Table).

The conservation and distribution of these novel MAPK domains in a group-specific manner could suggest that they are involved either in maintaining the structure of MAPKs, or in fine-tuning the selectivity towards substrates or protein interactors. This hypothesis is supported by homology structure prediction of selected MAPKs, which shows that domains 12, 13, 15, 15, and 17 are exposed to the solvent and could therefore provide an interaction surface. Meanwhile domain 14 is in a pocket (S4 Fig). Moreover, a computational prediction of phosphorylation sites, suggests that domains 15 and 16 contain amino acid residues susceptible to phosphorylation (S4 Table). Whether these residues are indeed phosphorylated, or if the identified novel domains are of relevance for the MAPKs structure and function, remains to be experimentally demonstrated and extends beyond the scope of this study.

Identification of MEK proteins across Viridiplantae

98 MEK genes were identified in the analyzed species. *B. distachyon* was the species with the highest number of MEK genes (12), followed by *A. thaliana* and *O. sativa*, with 10 and 9 genes, respectively. The algae *O. tauri* and *C. reindhardtii* are the species with the lowest number of MEK genes, having only one, each. The number of MEK genes encoded in the genome of the analyzed species suggest a gradual but constant gene MEK expansion in Viridiplantae (Table 4).

Table 4. MEKs Genes identified in different plant genomes

	**Specie**	**Lineage**	**MEKs gene number**
1	*Arabidopsis thaliana* L.	Angiosperm (Eudicot)	10
2	*Amaranthus hypochondriacus* L.	Angiosperm (Eudicot)	5
3	*Beta vulgaris* L.	Angiosperm (Eudicot)	5
4	*Vitis vinifera* L.	Angiosperm (Eudicot)	5
5	*Brachypodium distachyon* [L.] P. Beauv.	Angiosperm (Monocot)	12
6	*Oryza sativa* L.	Angiosperm (Monocot)	9
7	*Amborella trichopoda* Baill.	Angiosperm (Basal)	7
8	*Picea abies* [L] H. Karst.	Gymnosperm	7
9	*Pinus taeda* L.	Gymnosperm	3
10	*Azolla filiculoides* [Lam.]	Pteridophyta	6
11	*Salvinia cucullata* [Bory.]	Pteridophyta	4
12	*Isoetes echinospora* Durieu.	Lycophyta	4
13	*Selaginella moellendorffii*	Lycophyta	3
14	*Sphagnum fallax* H. Klinggr.	Bryophyta	6
15	*Marchantia polymorpha* L.	Bryophyta	3
16	*Physcomitrella patens* [Hedw.] Bruch & Schimp.	Bryophyta	7

17	*Chlamydomonas reindhardtii* P. A. Dang.	Algae	1
18	*Ostreococcus tauri* C. Courties & M.-J. Chrétiennot-Dinet	Algae	1

[illegible]npared with MAPKs, the proteins of the MEK group are on average smaller, with an average length of ~350 amino acids. however, the longest protein in this group, PtMEK4 is 1333 amino acids. On the other hand, the smallest protein is PaMEK3 from *P. abies* has a length of 133 amino acids. The arrangement of introns present in the MEK sequences ranges from 1 to 10. Unlike MAPK genes, which usually contain one or more introns, 27 of the identified MEK genes (> 30%) lack introns. The MEK sequences do not show an intron arrangement that characterizes the gene family, including genes with one (6 sequences); two (3); three (1); four (3); three (1); four (3); five (2); six (5); seven (17); eight (11); nine (9); and ten introns (1) (S1 Table). In addition, the analysis of *I. echinospora* transcriptome allowed the identification of four MEK sequences, but no information about the number of introns can be gathered from this data [75]. The molecular weight of the identified MEK proteins range from 5,877 Da (AhMEK3) to 74,002 Da (PtMEK4), while the isoelectric points range from 5.24 and 10.15 (S1 Table; S5 Fig).

Phylogenetic analysis

A multiple alignment of the retrieved MEK sequences was generated, this showed that MEK proteins are highly similar and that the 8 characteristic domains of MEK proteins are highly conserved, as well as the ATP-binding and MAPK-binding domains (S6 Fig). Ichimura *et al.*, classified MEK proteins into four groups, however this classification system was generated from angiosperm sequences only [17]. To avoid bias in the phylogeny reconstruction sequences retrieved from gymnosperms, seedless vascular plants, and non-vascular plants were included, as well as and the MEK protein Ste7 from *S. cerevisiae* as an outgroup to root the tree. The obtained ML topology shows that MEK proteins partition into five groups, named A-E (Fig 5).

Fig 5. Phylogenetic analysis of Viridiplantae MEK proteins. Maximum-likelihood tree reconstructed from amino acid sequences. The tree was rooted with the Ste7 protein from Saccharomyces cerevisiae. Bootstrap values from 1,000 replicates are indicated at nodes with

support values >90%. Each MEK group is indicated with a distinctive color. Different species are indicated with different shape and color.

MEK proteins are involved in a plethora of biological processes; for example, some members of group A, such as AtMEK1 and AtMEK2, are involved in responses to environmental stimuli [46,76]; while AtMEK6 participates in cell division [77]. The MEK proteins included in group B have an extended C-terminal with the conserved sequence E-[K/R]-L-V-H-V-V-E-[K/N]-L-[H/Q]-C-X-A-X_{1-4}-G-[I/V]-X-I-R-V. This group had been reported earlier [24] and includes AtMEK3, whose C-terminal extension is similar to the NTF domain, which is involved in the nuclear import of protein cargos [24,27,78,79]. Although the NTF domain is found in most eukaryotic lineages, its fusion to a MEK protein has only been observed in Viridiplantae [24,79]. AtMEK3 is the only member of the group B MEK proteins that has been characterized; it plays important roles in jasmonate [80] and blue light responses [81]. To the best of our knowledge, no biological function has been ascribed to any other member of the group, not even for the NTF domain *per se*, and therefore, the biological processes in which group B MEKs are involved remain obscured. The presence of the NTF domain in all group B MEKs, including sequences from angiosperms, gymnosperms, pteridophytes and bryophytes, but no algae, strongly suggests that the NTF–MEK domain combination occurred after the divergence of embryophytes, estimated around 500 MYA [27]. Moreover, its conservation in extant taxa might reflect an evolutionary advantage in photosynthetic eukaryotes [79] although biochemical or functional characterization of group B MEKs are needed to corroborate this hypothesis.

The available information about groups C and D MEKs is scarce, but some members from this clade are involved in hormone signaling pathways. For example, AtMEK4/5, which belong to group C, mediate ABA signaling pathways in response to biotic stress [45]. AtMEK7 and AtMEK9, which belong to group D, participate in polar transport of auxins [82], and in the response to ethylene [83], respectively. The novel group E proposed here contains sequences from bryophytes (*M. polymorpha, P. patens* and *S. fallax*), lycophytes (*S. moellendorffii*) pteridophytes (*A. filiculoides* and *S. cucullata*), gymnosperms (*P. abies*), and the early divergent angiosperm *A. trichopoda*. Interestingly, no group E MEKs were identified in monocot or dicotyledonous species, which might suggest that these genes

were lost prior to the Mesangiospermae origin, which diverged ~180 MYA [84]. Nonetheless, group E MEKs appears as a sister clade to group C and D, and this is well supported from bootstrap values (Fig 5).

Analysis of motifs and domains conserved in the MEK family

MEK proteins contain several conserved sequences to maintain the proper structure and function of these enzymes [85]. The MAPK-binding domain or docking-site (Table 5; S5 Table), is typically located towards the N-terminal end and its basic residues, lysine and arginine, interact with the acidic residues from the binding domain of MAPKs [74]. In addition, MEKs also contain an ATP-binding domain, and the activation domain (Table 5; S5 Table), where serine/threonine residues are prone to phosphorylation and are involved in signal transduction [39]. Some proteins, such as OsMEK7 or BdMEK7a, have insertions within its activation domain, with one of serine/threonine residue absent or located around 3 to 5 residues upstream of the canonical position (S5 Table). Other proteins, such as ScMEK2, lack completely the MAPK-binding domain (Fig 6; S5 Table). It has not been documented whether these genes are expressed nor if their gene products would exhibit MEK activity in MAP kinase cascades. If expressed, these proteins could represent neo or sub-functionalized versions arising from gene duplication events, or even act as a noncatalytic scaffolding proteins [27,86].

Fig 6. Canonical domains in Viridiplantae MEK proteins. The identification of the canonical MEK domains was performed with the MEME suite [55]. Sequence nomenclature follows two-letter code to indicate genus and species (see text).

Ta[illegible] 5. Consensus sequence of characteristic MEK domains.

Domain	Consensus sequence
I	G-X-[S/A/N]-[G/S]-G-X-V-X-[K/L]-[V/A]-X-H-[K/R]
II	P-X-[V/L]-V-X-[C/F]-[H/Y]-X_2-[F/Y]
III	[L/M]-E-[Y/F]-M-D-X-G-S-L-[A/E]
IV	[A/S]-X_6-L-X-G-L-X-Y-L-H
V	V-G-T-X_2-Y-M-S-P-E-R-[I/F]
VI	[G/S/A]-D-[I/V]-W-S-[L/F]-G-[L/V]-X_2-L-E
VII	[F/A]-S-X-E-[F/L]-[R/C]-X-F-[I/V]-X_2-C

VIII	[S/T]-[A/V]-X_2-L-L-X-H-P-F-[I/V/L]
ATP-binding	H-K-X_{3-8}-A-L-K-X_4-[N/D]-X-[D/E/Q]
MAPK-binding	[V/I]-H-R-D-[I/L]-K-P-[S/A]-N-L-L
Activation	V-S-X_5-[S/T]-[M/L]-[D/G/A]
NTF2-binding	E-[K/R]-[L/I]-[V/I]-H-V-V-E-[K/N]-L-[Q/H]-C

A scanning of the retrieved MEK sequences with the MEME suite confirmed the existence of eight domains, named I-VIII (Table 5; S5 Table). Domains I-IV are located prior the activation domain and are present in the vast majority of the MEK proteins analyzed, with the exceptions of PpMEK3a, PaMEK 6, and IeMEK10a, which lacks the domain I; VvMEK9, lacking domain II; VvMEK5, which lacks domains I and II; BdMEK9c and CrMEK6, which do not contain domain III; PaMEK10a, lacks domains I-III; and AhMEK3 and ScMEK2, which lack domain IV. On the other hand, domains V-VIII are located towards the C-terminal end, after the activation site. Domains V, and VI, are absent only in AfMEK6b; domain VII, is not present in VvMEK2, VvMEK9, IeMK10b, and IeMEKc; and domain VIII is not found in VvMEK2, VvMEK9, AmtrMEK6, PtMEK6b, AtMPEK8, and PaMEK6b. Additionally, six other sequences lack at least two domains PaMEK3 (V, VI and VIII); PaMEK4a (VII and VIII); PAMEK4B (V and VII); PaMEK6a and IeMEK6 (VII and VIII); and IeMEK10 (VII and VIII) (Fig 6; S5 Table). Nevertheless, the presence of these signature sequences could be used as a criterion to refine MEK homology searches or annotation.

Identification of novel and distinctive domains for each group of MEKs

MEK proteins include a MAPK binding domain with the consensus sequence [V/I]-H-R-D-[I/L]-K-P-[S/A]-N-L-L. This domain is involved in protein-protein interaction with its MAPK partner, and therefore is essential for MAPK signaling cascades [87]. A closer examination of this sequence reveals group-specific variations of the MAPK binding domain, which suggests specificity, at least to some level, in MEK selection towards its MAPK interactors, prompting the hypothesis that each MEK group might be involved in specific biological processes (Table 6).

Table 6. Variations in the sequence of the MAPK-binding motif in each group.

Group	Sequence
A	H-X_2-[R/K]-[H/R]-[I/V]-I-H-R-D-[I/L]-K-P-S-N-L-L
B	H-X-V-R-H-L-V-H-R-D-I-K-P-A-N-[L/M]-L
C	H-X_2-R-X-I-V-H-R-D-X-K-P-[S/A]-N-L-L
D	H-X_3-K-I-V-H-R-D-I-K-P-X-N-L-L
E	H-K-X_2-[H/N]-K-I-V-H-R-D-I-K-P-S-N-L-L

In addition to the canonical MEK domains, six new motifs spanning 10-18 amino acid residues were identified. Remarkably, these new motifs are group-specific and therefore can be used to quickly assign new MEK sequences to their respective ortholog group. These domains were named with consecutive cardinal numbers [9-14] (Fig 7; Table 7). With the exception of PaMEK6b, IeMEK6, PtMEK6a, and OtMEK2, all proteins from group A contain the domain 9, which is located towards the N-ter. Proteins from group B contain the domain 10 towards the N-terminal and domains 12 and 13 towards the C-terminal. MEKs from the C and E group contain domains 11 and 14, respectively, located towards the C-terminal of the protein. MEK proteins from the D group do not contain any domains in addition to the canonical ones (Fig 7; S6 Table). A computational analysis of post-translational modifications showed that all the putative novel domains contain potential phosphorylation sites, which might suggest that these domains are functionally relevant, possibly in the regulation of MEK activity; this hypothesis, however, requires experimental validation (S4 Table).

Fig 7. Identification of novel domains in Viridiplantae MEK proteins. The analysis of novel domains was performed with the MEME suite [55]. The combinatorial distribution of the novel domains is group specific, then it could facilitate future MEK identification and annotation. Sequence nomenclature follows a two-letter code to indicate genus and species (see text).

Table 7. Consensus sequence of novel MEKs domains.

Domain	Consensus sequence
9	[A/Q]-S-G-T-F-X-D-G-D-[L/I]-X-[L/V]-N-X_2-G
10	N-L-L-S-R-S-X_3-Y-N-[I/F]-N-E-X-G-[L/F]
11	L-P-[L/M]-P-X-R-X_2-D-X_{2-4}-S-L-A-V-P-L
12	M-L-[T/A]-[V/I]-H-Y-Y-[L/M]-L-F-[D/N]-G-X-D

13	G-[V/I]-X-I-R-S-G-S-F-[I/V]-V-G-X_2-F
14	[D/E]-P-P-X-P-P-X_3-[S/T]-P-X-F-X_2-F-I

Current knowledge on MEKK family

Plant MEKK are the least studied component of MAPK signaling cascades and therefore the available information for these proteins is scarce compared to the information available for MAPKs and MEKs [16,29,46,88]. The *Arabidopsis* genome encodes at least 80 genes distributed in three kinase subfamilies whose orthologs in animals have been shown to have MEKK activity: MAPKKK (**MAPK**/ERK **K**inase **K**inase, 21 members), ZIK (**Z**R1-**I**nteracting **K**inase, 11 members) and RAF (**R**apid **A**cceleration **F**ibrosarcoma, 48 members) [29]. Kinase activity has been confirmed for several members of the MAPKKK subfamily in plants, but no evidence has been reported for members of the ZIK subfamily [29,45,47,63]. CTR1 (Constitutive Triple Response1) and EDR1 (Enhanced Disease Resistance 1), which belong to the RAF subfamily, act primarily as negative regulators of MAP kinase modules [89–92], but no functional characterization has been made for other RAF members.

Plant MPKKKs have a variable MAP kinase domain [17]. Most proteins from the MEKK group have a regulatory domain and a serine/threonine residue at the activation site [18]. The proteins belonging to the MEKK group have the consensus sequence G-[T/S]-P-X-[F/Y/W]-M-A-P-E-V in the kinase domain [31,37], those from the ZIK group contain the sequence G-T-P-E-F-M-A-P-E-[L/V/M]-[Y/F/L], located towards the N-terminal end, meanwhile, members of the RAF group have the sequence G-T-X-X-[W/Y]-M-A-P-E. In addition, most proteins from the RAF group, have the kinase domain located towards the C-terminal end; while the N-terminal end contains a regulatory domain.

Conclussions

MAPK signaling cascades are conserved and widespread in eukaryotic organisms, where they are crucial in signal transduction in response to biotic, abiotic, and developmental cues [17,44]. Despite their involvement in several biological processes, and the previous efforts to elucidate the evolutionary paths of MAPKs diversification in plants, this has been limited by the intrinsic properties of the MAPK signaling cascade components, namely its functional redundance, high similarity at the

amino acid level, gene family expansion and contraction in several plant taxa, and the availability of sequenced plant genomes.

In the present work the phylogenetic relationships existing between MAPKs from 18 Viridiplantae species was analyzed. The analyzed species were selected to span the major Viridiplantae clades, ranging from Chlorophyta, i.e., green algae, up to angiosperms. Previous approaches to elucidate the evolutionary relationships of plant MAPKs were biased towards angiosperms, as the first plant sequenced genomes belonged to species from this clade [17]. Here we overcome this issue by including unicellular chlorophyta (*Ostreococcus tauri* and *Chlamydomonas reindhardtii*), bryophytes (*Marchantia polymorpha*, *Physcomitrella patens*, and *Sphagnum fallax*); the genome and transcriptome of *Selaginella moellendorffii* and *Isoetes tegetiformes*, respectively, both belonging to the lycophyte clade [75,93]; the recently released genomes of pteridophyte (*Azolla filiculoides* and *Salvinia cucullata*) [69]; and the gymnosperm genomes (*Picea abies* and *Pinus taeda*). As for the angiosperms we include basal species (*A. trichopoda*), and both monocots and dicots. This allows for a comprehensive and genome-wide exploration of MAPKs across Viridiplantae; and the consequent reconstruction of gene phylogenies to assess the diversification of the MAPK signaling cascades in plants. Our approach led to the identification of 189 and 98 MAPK and MEK genes, respectively, and therefore stressed the need for a heuristic nomenclature system. We expand on the system proposed by Hamel *et al.* (2006) to name MAPK genes according to their Arabidopsis orthologs and signature sequences, which typify MAPK genes in distinctive groups. The proposed nomenclature might be used as a practical tool to aid the identification of novel MAPK genes in additional plant genomes, and even during genome annotation projects. The reconstructed ML trees exhibit a well resolved and supported topology, and therefore is useful to recognize the most likely Arabidopsis ortholog of the retrieved MAPK sequences.

Orthology based nomenclature systems provide functional insights for each MAPK clade [27,34,94], and thus, some of the novel domains identified in this work could be correlated to substrate, activator, or inhibitor specificity; as well as to protein-protein interaction. It is likely that some of the conclusions reached, or the number of identified sequences in some of the analyzed species, might need to be

adjusted as new genome versions are updated or reannotated. Also, the inclusion of more genomes, specifically from plant clades that are still underrepresented, such as lycophyte or gymnosperm, might help refine the reconstructed phylogenies, but international collaborative projects, such as the 10KP will overcome this gap in the near future [95].

Acknowledgements

The authors thank Gustavo Delgado-Prudencio and Adrian Martínez-Santana (graduate students in the Instituto de Biotecnología-UNAM) for their insightful suggestions and help with phylogenetic analysis. The technical assistance from MTI Juan Manuel Hurtado-Ramírez and MEM David Santiago Castañeda-Carreón (Unidad de Cómputo del Instituto de Biotecnología-UNAM) is also greatly acknowledged

References

1. Bigeard J, Hirt H. Nuclear signaling of plant MAPKs. Front Plant Sci. 2018;9(April):1–18.

2. Olsen J V., Mann M. Status of large-scale analysis of posttranslational modifications by mass spectrometry. Mol Cell Proteomics. 2013;12(12):3444–52.

3. Bigeard J, Rayapuram N, Pflieger D, Hirt H. Phosphorylation-dependent regulation of plant chromatin and chromatin-associated proteins. Proteomics. 2014;14(19):2127–40.

4. Ichimura K, Mizoguchi T, Yoshida R, Yuasa T, Shinozaki K. Various abiotic stresses rapidly activate Arabidopsis MAP. 2000;24:655–65.

5. Mishra NS, Tuteja R, Tuteja N. Signaling through MAP kinase networks in plants. Arch Biochem Biophys. 2006;452(1):55–68.

6. Andreasson E, Ellis B. Convergence and specificity in the Arabidopsis MAPK nexus. Trends Plant Sci. 2010;15(2):106–13.

7. González Besteiro MA, Ulm R. Phosphorylation and stabilization of Arabidopsis MAP kinase phosphatase 1 in response to UV-B stress. J Biol Chem. 2013;288(1):480–6.

8. Kersten B, Agrawal GK, Durek P, Neigenfind J, Schulze W, Walther D, et al. Plant phosphoproteomics: An update. Proteomics. 2009;9(4):964–88.

9. Schweighofer A, Hirt H, Meskiene I. Plant PP2C phosphatases: Emerging functions in stress signaling. Trends Plant Sci. 2004;9(5):236–43.

10. Tuteja N. Signaling through G protein coupled receptors. Plant Signal Behav [Internet]. 2009;4(10):942–7. Available from: http://www.tandfonline.com/doi/abs/10.4161/psb.4.10.9530

11. Venerando A, Cesaro L, Pinna LA. From phosphoproteins to phosphoproteomes: a historical account. FEBS J. 2017;284(13):1936–51.

12. Stone JM, Walker JC. Plant Protein Kinase Families and Signal Transduction’. 1995;451–7.

13. Dardick C, Chen J, Richter T, Ouyang S, Ronald P. The rice kinase database. A phylogenomic database for the rice kinome. Plant Physiol. 2007;143(2):579–86.

14. The Arabidopsis Genome Initiative. Analysis of the genome sequence of Arabidopsis thaliana. Nature. 2000;408(December):796–815.

15. Craig Venter J, Adams MD, Myers EW, Li PW, Mural RJ, Sutton GG, et al. The sequence of the human genome. Science (80-). 2001;291(5507):1304–51.

16. Colcombet J, Hirt H. *Arabidopsis* MAPKs: a complex signalling network involved in multiple biological processes. Biochem J [Internet]. 2008;413(2):217–26. Available from: http://biochemj.org/lookup/doi/10.1042/BJ20080625

17. Ichimura K, Shinozaki K, Tena G, Sheen J, Henry Y, Champion A, et al. Mitogen-activated protein kinase cascades in plants: A new nomenclature. Trends Plant Sci. 2002;7(7):301–8.

18. Wang J, Pan C, Wang Y, Ye L, Wu J, Chen L, et al. Genome-wide identification of MAPK, MAPKK, and MAPKKK gene families and transcriptional profiling analysis during development and stress response in cucumber. BMC Genomics [Internet]. 2015;16(1). Available from: ???

19. Tena G, Asai T, Chiu W-L, Sheen J. Plant mitogen-activated protein kinase signaling cascades. Curr Opin Plant Biol [Internet]. 2001;4(5):392–400. Available from: http://www.sciencedirect.com/science/article/pii/S1369526600001916

20. Singh A, Nath O, Singh S, Kumar S, Singh IK. Genome-wide identification of the MAPK gene family in chickpea and expression analysis during development and stress response. Plant Gene [Internet]. 2018;13(May 2017):25–35. Available from: https://doi.org/10.1016/j.plgene.2017.12.001

21. Nakagami H, Pitzschke A, Hirt H. Emerging MAP kinase pathways in plant stress signalling. 2005;10(7).

22. Zhang S, Klessig DF. MAPK cascades in plant defense signaling. 2001;6(11):520–7.

23. Pitzschke A, Schikora A, Hirt H. MAPK cascade signalling networks in plant defence. Curr Opin Plant Biol. 2009;

24. Dóczi R, ökrész L, Romero AE, Paccanaro A, Bögre L. Exploring the evolutionary path of plant MAPK networks. Trends Plant Sci. 2012;17(9):518–25.

25. López-Bucio JS, Dubrovsky JG, Raya-González J, Ugartechea-Chirino Y, López-Bucio J, De Luna-Valdez LA, et al. Arabidopsis thaliana mitogen-activated protein kinase 6 is involved in seed formation and modulation of primary and lateral root development. J Exp Bot. 2014;65(1):169–83.

26. Wu P, Wang W, Li Y, Hou X. Divergent evolutionary patterns of the MAPK cascade genes in Brassica rapa and plant phylogenetics. Hortic Res. 2017;4(June).

27. Hamel LP, Nicole MC, Sritubtim S, Morency MJ, Ellis M, Ehlting J, et al. Ancient signals: comparative genomics of plant MAPK and MAPKK gene families. Trends Plant Sci. 2006;11(4):192–8.

28. Chen J, Piao Y, Liu Y, Li X, Piao Z. Genome-wide identification and expression analysis of chitinase gene family in Brassica rapa reveals its role in clubroot resistance. Plant Sci. 2018;270:257–67.

29. Chardin C, Krapp A, Schenk ST, Hirt H, Colcombet J. Review: Mitogen-Activated Protein Kinases in nutritional signaling in Arabidopsis. Plant Sci [Internet]. 2017;260(March):101–8. Available from: http://dx.doi.org/10.1016/j.plantsci.2017.04.006

30. Luo J, Wang X, Feng L, Li Y, He JX. The mitogen-activated protein kinase kinase 9 (MKK9) modulates nitrogen acquisition and anthocyanin accumulation under nitrogen-limiting condition in Arabidopsis. Biochem Biophys Res Commun [Internet]. 2017;487(3):539–44. Available from: http://dx.doi.org/10.1016/j.bbrc.2017.04.065

31. Jonak C, Ökrész L, Bögre L, Hirt H. Complexity, cross talk and integration of plant MAP kinase signalling. Curr Opin Plant Biol. 2002;5(5):415–24.

32. Popescu S, Popescu G, Bachan S, Zhang Z, Gerstein M, Snyder M, et al. MAPK target networks in Arabidopsis thaliana revealed using functional protein microarrays. Genes Dev [Internet]. 2009;23:80–92. Available from: http://genesdev.cshlp.org/content/23/1/80.short

33. Çakır B, Kılıçkaya O. Mitogen-activated protein kinase cascades in Vitis vinifera. Front Plant Sci [Internet]. 2015;6(July). Available from: http://journal.frontiersin.org/Article/10.3389/fpls.2015.00556/abstract

34. Mohanta TK, Arora PK, Mohanta N, Parida P, Bae H. Identification of new members of the MAPK gene family in plants shows diverse conserved domains and novel activation loop variants. BMC Genomics. 2015;16(1):1–20.

35. Zhan H, Yue H, Zhao X, Wang M, Song W, Nie X. Genome-wide identification and analysis

of MAPK and MAPKK gene families in bread wheat (Triticum aestivum L.). Genes (Basel). 2017;8(10).

36. Wang M, Yue H, Feng K, Deng P, Song W, Nie X. Genome-wide identification, phylogeny and expressional profiles of mitogen activated protein kinase kinase kinase (MAPKKK) gene family in bread wheat (Triticum aestivum L.). BMC Genomics [Internet]. 2016;17(1). Available from: http://dx.doi.org/10.1186/s12864-016-2993-7

37. Rao KUPR, Richa TA, Kumar KU, Raghuram BA, Sinha ALOKKR. In Silico Analysis Reveals 75 Members of Mitogen-Activated Protein Kinase Kinase Kinase Gene Family in Rice. 2010;(April):139–53.

38. Chen L, Hu W, Tan S, Wang M, Ma Z, Zhou S, et al. Genome-Wide Identification and Analysis of MAPK and MAPKK Gene Families in Brachypodium distachyon. PLoS One. 2012;7(10):18–9.

39. Jiang M, Chu Z. Comparative analysis of plant MKK gene family reveals novel expansion mechanism of the members and sheds new light on functional conservation. BMC Genomics. 2018;19(1):1–18.

40. Liu Y, Zhang D, Wang L, Li D. Genome-Wide Analysis of Mitogen-Activated Protein Kinase Gene Family in Maize. Plant Mol Biol Report. 2013;31(6):1446–60.

41. Zhang S, Xu R, Luo X, Jiang Z, Shu H. Genome-wide identification and expression analysis of MAPK and MAPKK gene family in Malus domestica. Gene [Internet]. 2013;531(2):377–87. Available from: http://dx.doi.org/10.1016/j.gene.2013.07.107

42. Kong F, Wang J, Cheng L, Liu S, Wu J, Peng Z, et al. Genome-wide analysis of the mitogen-activated protein kinase gene family in Solanum lycopersicum. Gene [Internet]. 2012;499(1):108–20. Available from: http://dx.doi.org/10.1016/j.gene.2012.01.048

43. Zhou H, Ren S, Han Y, Zhang Q, Qin L, Xing Y. Identification and analysis of mitogen-activated protein kinase (MAPK) cascades in fragaria vesca. Int J Mol Sci. 2017;18(8).

44. Hirt H. Multiple roles of MAP kinases in plant signal transduction. Trends Plant Sci. 1997;2(1):11–4.

45. Asai T, Tena G, Plotnikova J, Willmann MR, Chiu WL, Gomez-Gomez L, et al. Map kinase signalling cascade in Arabidopsis innate immunity. Nature. 2002;415(6875):977–83.

46. Teige M, Scheikl E, Eulgem T, Doczi R, Ichimura K, Shinozaki K, et al. The MKK2 pathway mediates cold and salt stress signaling in Arabidopsis. Mol Cell [Internet]. 2004;15(1):141–52. Available from: http://www.ncbi.nlm.nih.gov/entrez/query.fcgi?cmd=Retrieve&db=PubMed&dopt=Citation&list_uids=15225555

47. Wang H, Ngwenyama N, Liu Y, Walker JC, Zhang S. Stomatal Development and Patterning Are Regulated by Environmentally Responsive Mitogen-Activated Protein Kinases in Arabidopsis. Plant Cell Online [Internet]. 2007;19(1):63–73. Available from: http://www.plantcell.org/cgi/doi/10.1105/tpc.106.048298

48. Johnson LS, Eddy SR, Portugaly E. Hidden Markov model speed heuristic and iterative HMM search procedure. BMC Bioinformatics. 2010;11.

49. Katoh K, Rozewicki J, Yamada KD. MAFFT online service: multiple sequence alignment, interactive sequence choice and visualization. Brief Bioinform [Internet]. 2017;(May):1–7. Available from: http://academic.oup.com/bib/article/doi/10.1093/bib/bbx108/4106928/MAFFT-online-service-multiple-sequence-alignment

50. Kuraku S, Zmasek CM, Nishimura O, Katoh K. aLeaves facilitates on-demand exploration of metazoan gene family trees on MAFFT sequence alignment server with enhanced interactivity. Nucleic Acids Res. 2013;41(Web Server issue):22–8.

51. Waterhouse AM, Procter JB, Martin DMA, Clamp M, Barton GJ. Jalview Version 2-A multiple sequence alignment editor and analysis workbench. Bioinformatics. 2009;25(9):1189–91.

52. Abascal F, Zardoya R, Posada D. ProtTest: Selection of best-fit models of protein evolution. Bioinformatics. 2005;21(9):2104–5.

53. Trifinopoulos J, Nguyen L, Haeseler A Von, Minh BQ. W-IQ-TREE : a fast online phylogenetic tool for maximum likelihood analysis. 2016;44(April):232–5.

54. Letunic I, Bork P. Interactive Tree of Life (iTOL) v4: Recent updates and new developments. Nucleic Acids Res. 2019;47(W1):256–9.

55. Bailey TL, Boden M, Buske FA, Frith M, Grant CE, Clementi L, et al. MEME S UITE : tools for motif discovery and searching. 2009;37(May):202–8.

56. Dou Y, Yao B, Zhang C. PhosphoSVM: Prediction of phosphorylation sites by integrating various protein sequence attributes with a support vector machine. Amino Acids. 2014;46(6):1459–69.

57. Crooks G, Hon G, Chandonia J, Brenner S. NCBI GenBank FTP Site\nWebLogo: a sequence logo generator. Genome Res [Internet]. 2004;14:1188–90. Available from: ftp://ftp.ncbi.nih.gov/genomes/Bacteria

58. Roy A, Kucukural A, Zhang Y. I-TASSER: A unified platform for automated protein structure and function prediction. Nat Protoc. 2010;5(4):725–38.

59. Wang B, Qin X, Wu J, Deng H, Li Y, Yang H, et al. Analysis of crystal structure of Arabidopsis MPK6 and generation of its mutants with higher activity. Sci Rep. 2016;6(April):1–13.

60. Dolan L, U BS. Root pattern : Shooting in the dark ? 1998;9.

61. Raven JA, Edwards D. Roots : evolutionary origins and biogeochemical significance. 2001;52(March).

62. Kovtun Y, Chiu W, Tena G, Sheen J. Functional analysis of oxidative stress-activated mitogen-activated protein kinase cascade in plants. 1999;

63. Takahashi F, Yoshida R, Ichimura K, Mizoguchi T, Seo S, Yonezawa M, et al. The Mitogen-Activated Protein Kinase Cascade MKK3-MPK6 Is an Important Part of the Jasmonate Signal Transduction Pathway in Arabidopsis. Plant Cell Online [Internet]. 2007;19(3):805–18. Available from: http://www.plantcell.org/cgi/doi/10.1105/tpc.106.046581

64. Lewis TS, Shapiro PS, Ahn NG. Signal Transduction through MAP Kinase Cascades. 1993;

65. Rasmussen MW, Roux M, Petersen M, Mundy J. MAP Kinase Cascades in Arabidopsis Innate Immunity. Front Plant Sci [Internet]. 2012;3(July):1–6. Available from: http://journal.frontiersin.org/article/10.3389/fpls.2012.00169/abstract

66. Calderini O, Bögre L, Vicente O, Binarova P, Heberle-Bors E, Wilson C. A cell cycle regulated MAP kinase with a possible role in cytokinesis in tobacco cells. J Cell Sci [Internet]. 1998;111 (Pt 2:3091–100. Available from: http://www.ncbi.nlm.nih.gov/pubmed/9739082

67. Schaffer R, Landgraf J, Accerbi M, Simon V, Larson M, Wisman E. Microarray Analysis of Diurnal and Circadian-Regulated Genes in Arabidopsis. 2001;13(January):113–23.

68. Miguel A, Bailly C, Boudsocq M, Resentini F, Puyaubert J, Baudouin E. The MPK8-TCP14 pathway promotes seed germination in Arabidopsis. 2019;677–92.

69. Li F, Brouwer P, Carretero-paulet L, Cheng S, Vries J De, Delaux P, et al. Fern genomes elucidate land plant evolution and cyanobacterial symbioses. Nat Plants [Internet]. 2018;4(July). Available from: https://doi.org/10.1038/s41477-018-0188-8

70. Leebens-Mack JH, Barker MS, Carpenter EJ, Deyholos MK, Gitzendanner MA, Graham SW, et al. One thousand plant transcriptomes and the phylogenomics of green plants. Nature. 2019;574(7780):679–85.

71. Panchy N, Lehti-Shiu M, Shiu SH. Evolution of gene duplication in plants. Plant Physiol. 2016;171(4):2294–316.

72. Van De Peer Y, Mizrachi E, Marchal K. The evolutionary significance of polyploidy. Nat Rev Genet [Internet]. 2017;18(7):411–24. Available from: http://dx.doi.org/10.1038/nrg.2017.26

73. Garrido E, Voß U, Müller P, Castillo-lluva S, Kahmann R. The induction of sexual development and virulence in the smut fungus Ustilago maydis depends on Crk1 , a novel MAPK protein. 2004;7:3117–30.

74. Tanoue T, Adachi M, Moriguchi T, Nishida E. A conserved docking motif in MAP kinases common to substrates, activators and regulators. Nat Cell Biol. 2000;2(2):110–6.

75. Hetherington AJ, Emms DM, Kelly S, Dolan L. Gene expression data support the hypothesis that Isoetes rootlets are true roots and not modified leaves. Sci Rep [Internet]. 2020 Dec 9;10(1):21547. Available from: https://doi.org/10.1038/s41598-020-78171-y

76. Helfer A, Hatzimasoura E, Serazetdinova L, Rios G, Teige M, Koncz C, et al. The Arabidopsis MAP kinase kinase MKK1 participates in defence responses to the bacterial elicitor flagellin. 2006;485–98.

77. Kosetsu K, Matsunaga S, Nakagami H, Colcombet J, Sasabe M, Soyano T, et al. The MAP Kinase MPK4 Is Required for Cytokinesis in Arabidopsis thaliana. 2010;22(November):3778–90.

78. Quimby BB, Wilson CA, Corbett AH. The Interaction Between Ran and NTF2 is Required for Cell Cycle Progression. 2000;11(August):2617–29.

79. Nicole MC, Hamel LP, Morency MJ, Beaudoin N, Ellis BE, Séguin A. MAP-ping genomic organization and organ-specific expression profiles of poplar MAP kinases and MAP kinase kinases. BMC Genomics. 2006;7:1–22.

80. Yuasa T, Ichimura K, Mizoguchi T, Shinozaki K. Oxidative Stress Activates ATMPK6 , an Arabidopsis Homologue of MAP Kinase. 2001;42(9):1012–6.

81. Sethi V, Raghuram B, Sinha AK, Chattopadhyay S. A Mitogen-Activated Protein Kinase

Cascade Module, MKK3-MPK6 and MYC2, Is Involved in Blue Light-Mediated Seedling Development in Arabidopsis. Plant Cell [Internet]. 2014;26(8):3343–57. Available from: http://www.plantcell.org/cgi/doi/10.1105/tpc.114.128702

82. Dai Y. Increased Expression of MAP KINASE KINASE7 Causes Deficiency in Polar Auxin Transport and Leads to Plant Architectural Abnormality in Arabidopsis. Plant Cell Online [Internet]. 2006;18(2):308–20. Available from: http://www.plantcell.org/cgi/doi/10.1105/tpc.105.037846

83. Yoo S, Sheen J. MAPK signaling in plant hormone ethylene signal transduction. 2008;3(10):848–9.

84. Smith SA, Beaulieu JM, Donoghue MJ. An uncorrelated relaxed-clock analysis suggests an earlier origin for fl owering plants. 2010;107(13):5897–902.

85. Goyal RK, Tulpan D, Chomistek N, Fundora DG, West C, Ellis BE, et al. Analysis of MAPK and MAPKK gene families in wheat and related Triticeae species. 2018;1–26.

86. Whitmarsh AJ, Davis RJ. Structural organization of MAP- kinase signaling modules by scaffold proteins in yeast and mammals. 1998;0004(December):21407–10.

87. Oh C, Martin GB. Tomato 14-3-3 Protein TFT7 Interacts with a MAP Kinase Kinase to Regulate Immunity-associated Programmed Cell Death Mediated by Diverse Disease Resistance Proteins * □. 2011;286(16):14129–36.

88. Xu J, Zhang S. Mitogen-activated protein kinase cascades in signaling plant growth and development. Trends Plant Sci [Internet]. 2014;1–9. Available from: http://dx.doi.org/10.1016/j.tplants.2014.10.001

89. Bigeard J, Colcombet J, Hirt H. Signaling mechanisms in pattern-triggered immunity (PTI). Mol Plant [Internet]. 2015;8(4):521–39. Available from: http://dx.doi.org/10.1016/j.molp.2014.12.022

90. Mithoe SC, Ludwig C, Pel MJ, Cucinotta M, Casartelli A, Mbengue M, et al. Attenuation of pattern recognition receptor signaling is mediated by a MAP kinase kinase kinase . EMBO Rep. 2016;17(3):441–54.

91. Zhao C, Nie H, Shen Q, Zhang S, Lukowitz W, Tang D. EDR1 Physically Interacts with MKK4/MKK5 and Negatively Regulates a MAP Kinase Cascade to Modulate Plant Innate Immunity. PLoS Genet. 2014;10(5).

92. Yoo SD, Cho YH, Tena G, Xiong Y, Sheen J. Dual control of nuclear EIN3 by bifurcate MAPK cascades in C 2H4 signalling. Nature. 2008;451(7180):789–95.

93. Banks JA, Nishiyama T, Hasebe M, Bowman JL, Gribskov M, DePamphilis C, et al. The selaginella genome identifies genetic changes associated with the evolution of vascular plants. Science (80-). 2011;332(6032):960–3.

94. Aravind L, Koonin E V. Towards understanding the first genome sequence of a crenarchaeon by genome annotation using clusters of orthologous groups of proteins (COGs) Darren A Natale , Uma T Shankavaram , Michael Y Galperin , Yuri I Wolf ,. 2000;1–19.

95. Cheng S, Melkonian M, Smith SA, Brockington S, Archibald JM, Delaux PM, et al. 10KP: A phylodiverse genome sequencing plan. Gigascience. 2018;7(3):1–9.

Supporting information

S1 Fig. Protein alignment of representative MAPK proteins from selected plant species. Twenty proteins representing the diversity of MAPKs in Viridiplantae were aligned using the MAFFT software [49] and visualized in Jalview [51]. The numerals I to XI indicate the conserved (canonical) kinase domains; the TXY, and CD labels indicate the activation site and docking domain, respectively.

S2 Fig. Distribution of the different activation domains In MAPK proteins. Most proteins from the A, B, and C group contain TEY activation motif, while proteins from the group D contain TDY

activation motif. Group E exhibits a high variability of activation motifs, specially in early divergent plant species.

S3 Fig. Biochemical characteristics of MAPK groups. Boxplots showing **a)** Molecular weight, **b)** Protein length, and **c)** Theorical isoelectric point of MAPK proteins according to their group. The boxplot indicates the median line, first and third quartiles. Outliers that are 1.5 above the upper quartile or below the lower quartile are indicated as points.

S4 Fig. Putative localization of the novel domains in the MAPK protein structure. The protein structures were obtained from homology modelling [58] and visualized in PyMol (The PyMOL Molecular Graphics System, Version 2.0 Schrödinger, LLC.). Each domain is presented as a sequence logo, obtained from the multiple sequence alignment of all the retrieved sequences (57), and highlighted in the protein structure with a distinctive color.

S5 Fig. Biochemical characteristics of MEK groups. Boxplots showing **a)** Molecular weight, **b)** Protein length, and **c)** Theorical isoelectric point of MEK proteins according to their group. The boxplot indicates the median line, first and third quartiles. Outliers that are 1.5× above the upper quartile or below the lower quartile are indicated as points.

S6 Fig. Protein alignment of representative MEK proteins from different plant species. Twenty proteins representing the diversity of MEKs across Viridiplantae were aligned using the MAFFT software [49] and visualized in Jalview [51]. The numerals I to VIII represent the canonical domains; the ATP-binding and MAPK-binding domain are also indicated.

S1 Table. List of the MAPK and MEK proteins identified in the genomes of selected species.

S2 Table. Sequences of the canonical domains in the MAPK family.

S3 Table. Sequences of the novel domains identified in the MAPK family.

S4 Table. Computational prediction of phosphorylation sites of the novel domains identified in the MAPK and MEK families.

S5 Table. Sequences of the canonical domains in the MEK family.

S6 Table. Sequences of the novel domains identified in the MEK family.

S1 File. Multiple sequence alignment of MAPK proteins. (TXT)

S2 File. Multiple sequence alignment of MEK proteins. (TXT)

S3 File. Newick format of the structure of the phylogenetic tree of MAPK proteins. (TXT)

S4 File. Newick format of the structure of the phylogenetic tree of MEK proteins. (TXT)

Figure 1

Click here to access/download;Figure;Fig1.tif

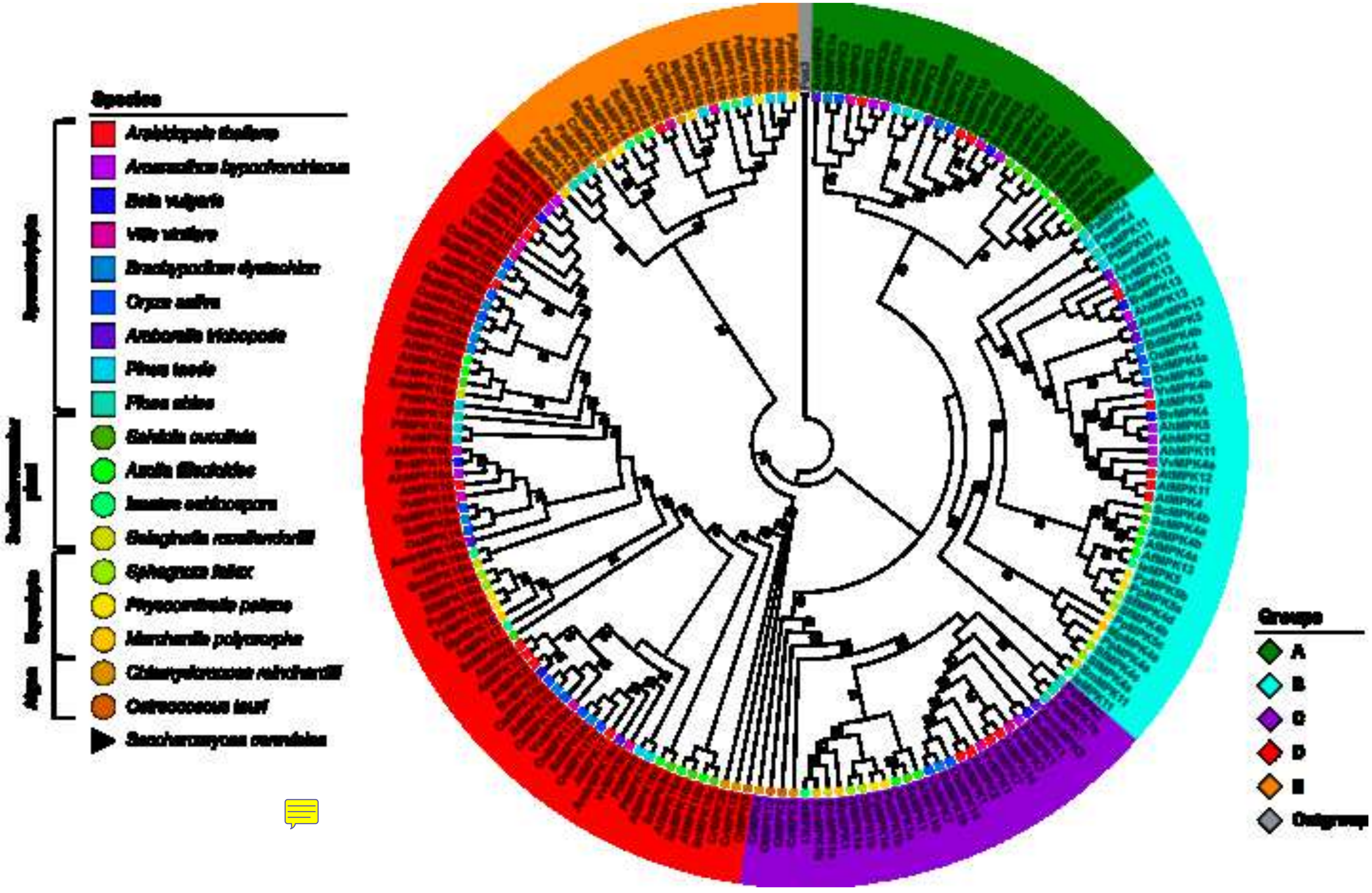

Figure 2

Click here to access/download;Figure;Fig2.tif

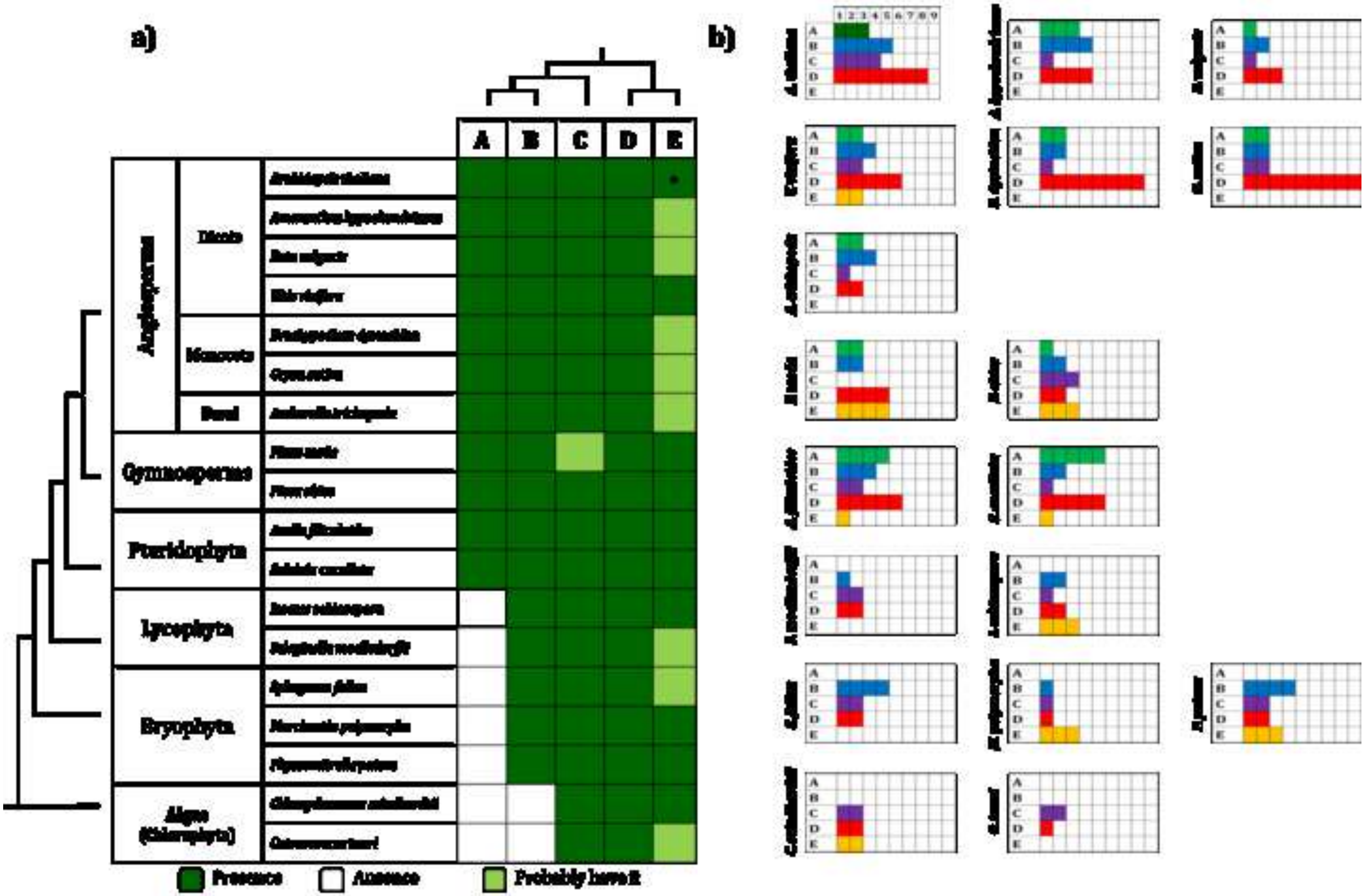

Figure 3

Click here to access/download;Figure;Fig3.tif

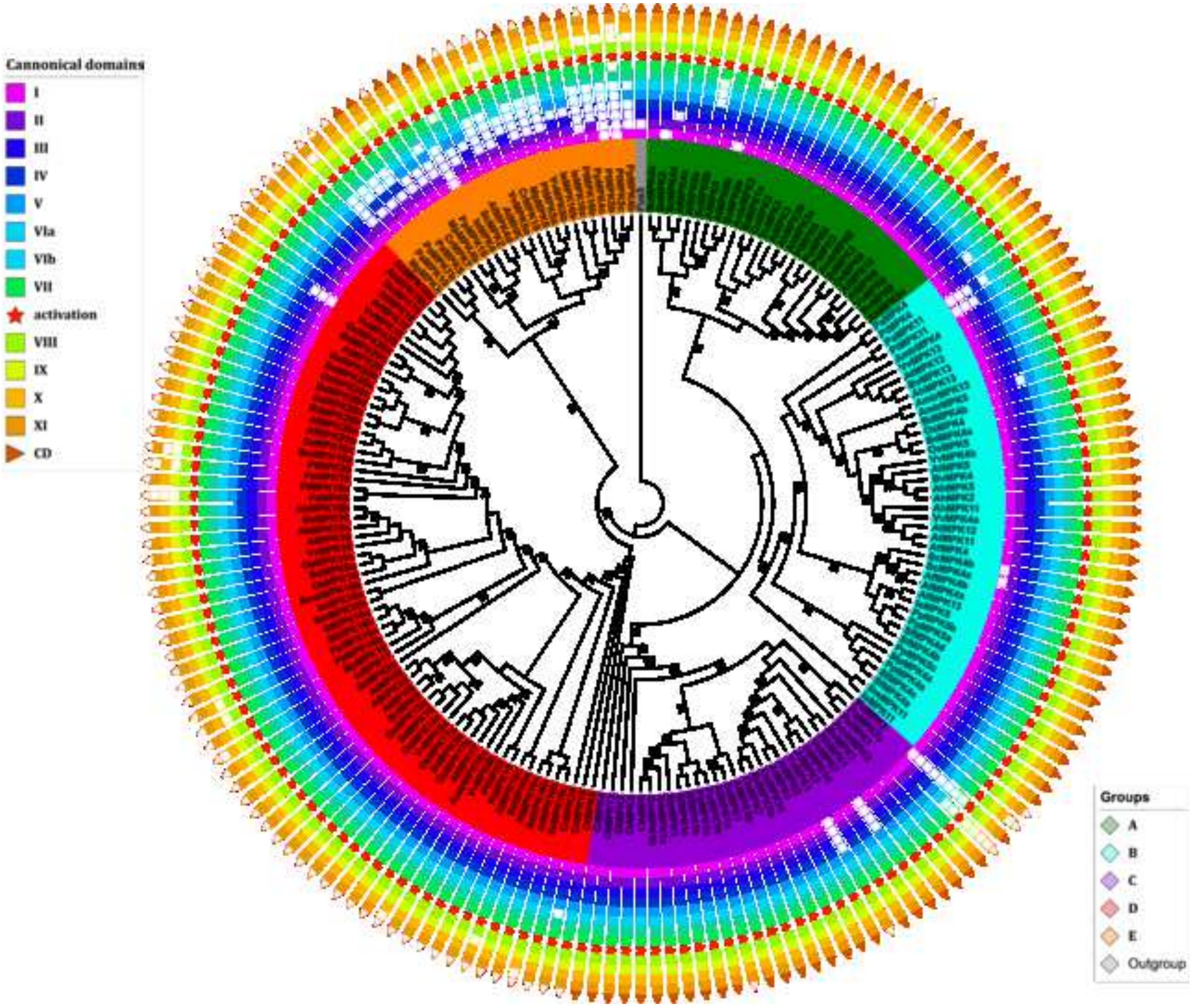

Figure 4

Click here to access/download;Figure;Fig4.tif

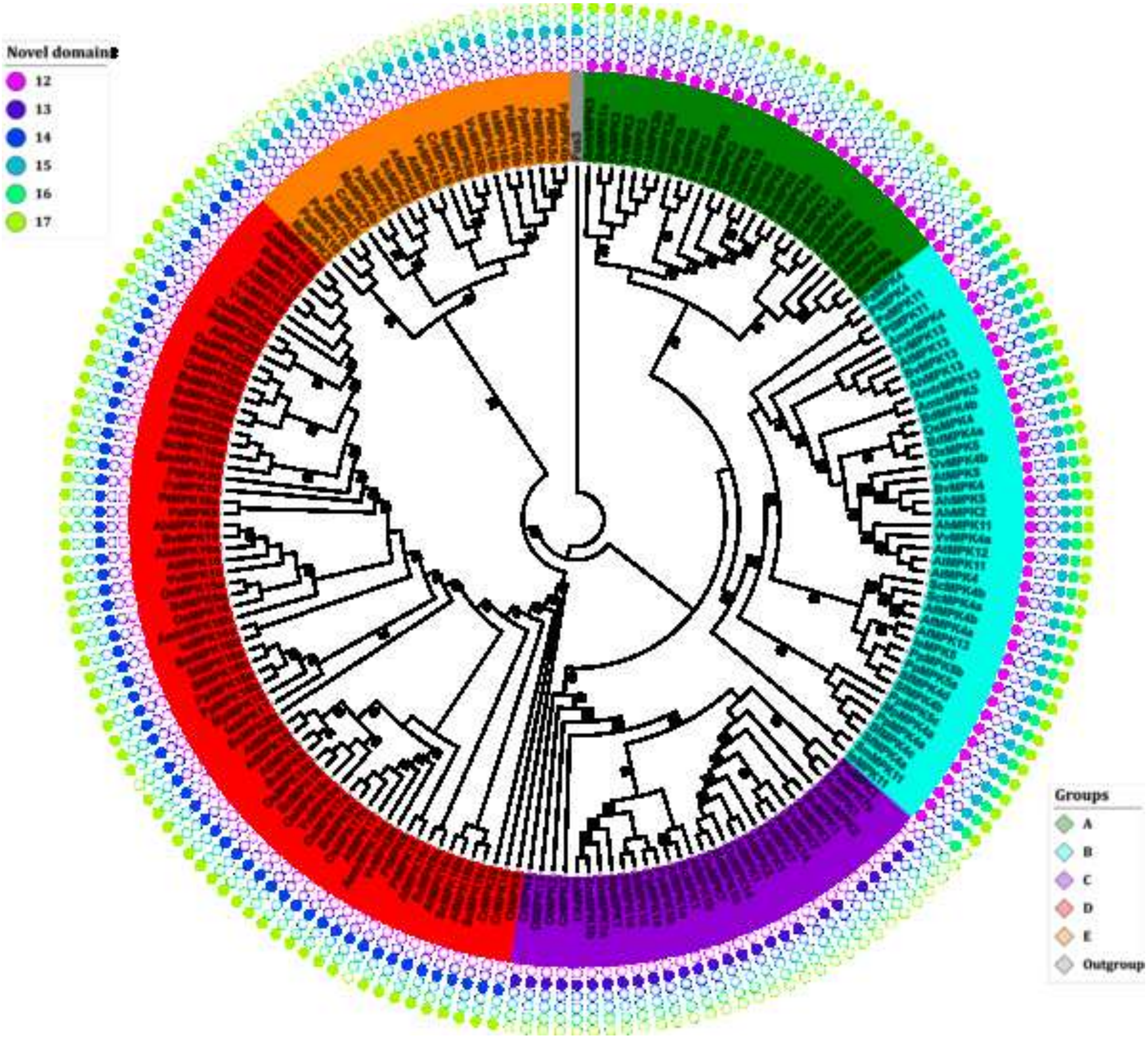

Figure 5

Click here to access/download;Figure;Fig5.tif

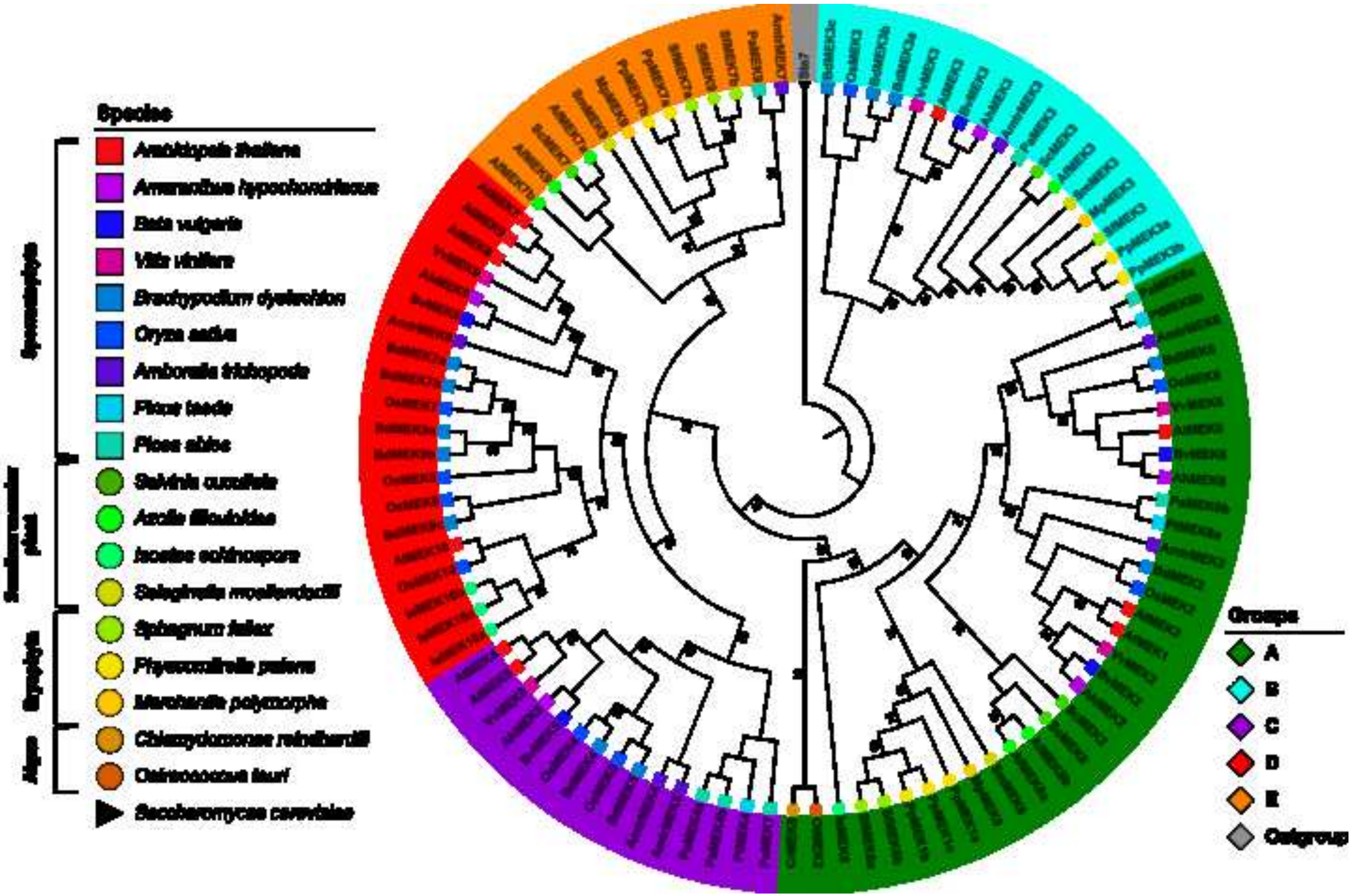

Figure 6

Click here to access/download;Figure;Fig6.tif

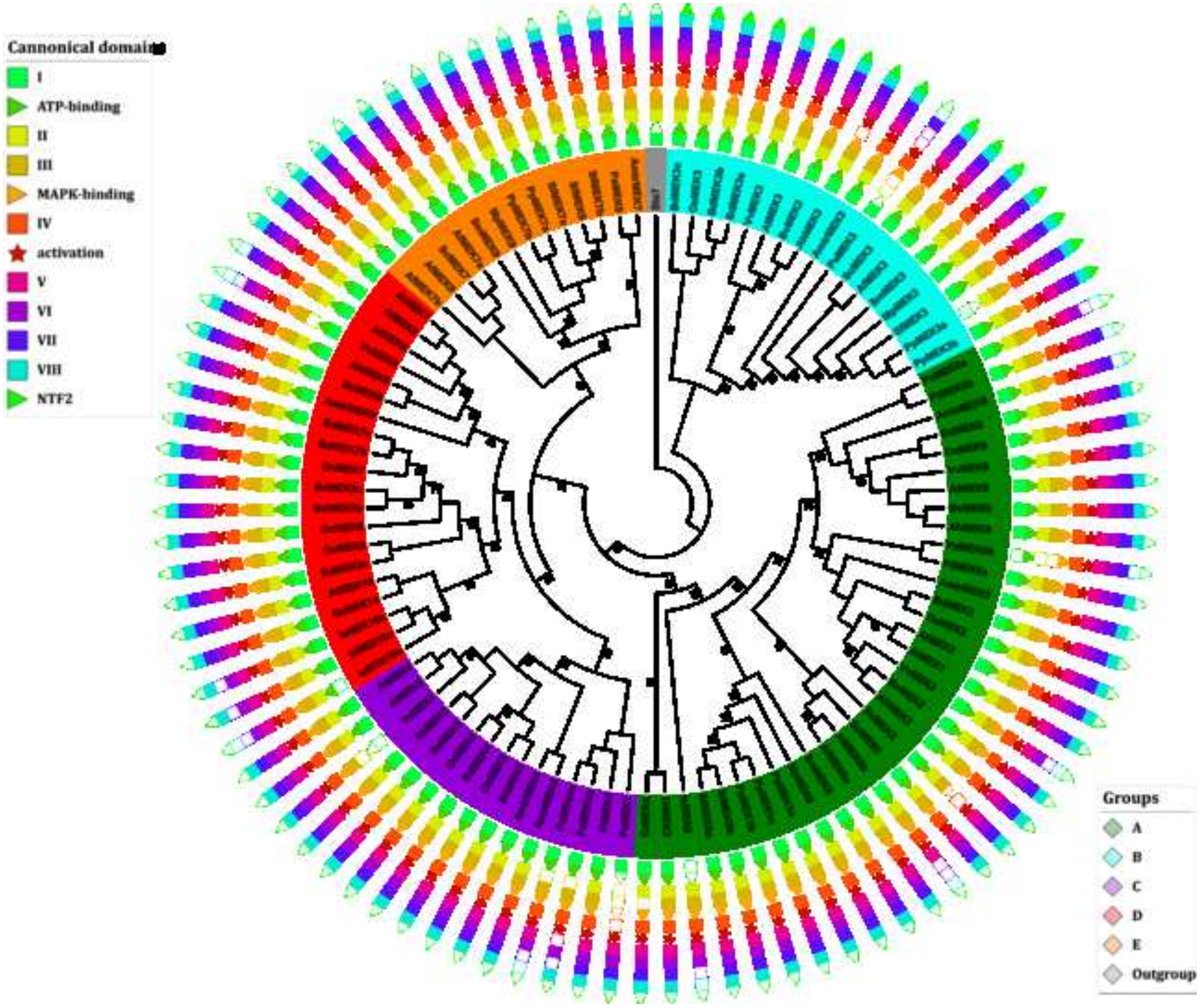

Figure 7 Click here to access/download;Figure;Fig7.tif

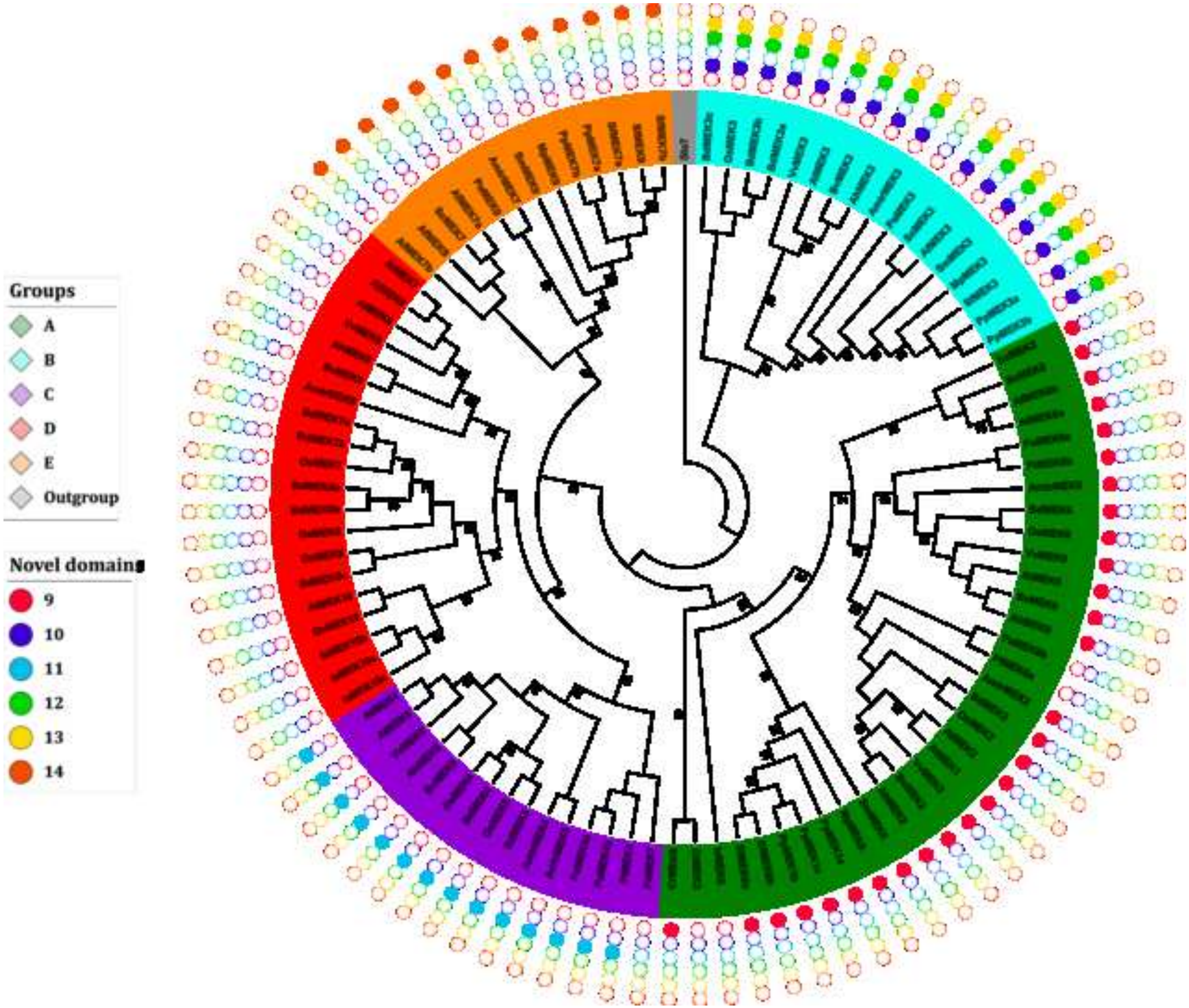

Click here to access/download
Supporting Information
S1 Fig.tif

Click here to access/download
Supporting Information
S2 Fig.tif

Click here to access/download
Supporting Information
S3 Fig.tif

Click here to access/download
Supporting Information
S4 Fig.tif

Click here to access/download
Supporting Information
S5 Fig.tif

Click here to access/download
Supporting Information
S6 Fig.tif

Click here to access/download
Supporting Information
S1 Table.xlsx

Click here to access/download
Supporting Information
S2 Table.xlsx

Click here to access/download
Supporting Information
S3 Table.xlsx

Click here to access/download
Supporting Information
S4 Table.xlsx

Click here to access/download
Supporting Information
S5 Table.xlsx

Click here to access/download
Supporting Information
S6 Table.xlsx

Click here to access/download
Supporting Information
S1 File.txt

Click here to access/download
Supporting Information
S2 File.txt

Click here to access/download
Supporting Information
S3 File.txt

Click here to access/download
Supporting Information
S4 File.txt
